# Supplementary material for: NFκB and NLRP3/NLRC4 inflammasomes regulate differentiation, activation and functional properties of monocytes in response to distinct SARS-CoV-2 proteins
Source: Nat Commun. 2024 Mar 7;15:2100. doi: 10.1038/s41467-024-46322-8 (PMC10920883; doi:10.1038/s41467-024-46322-8)
Supplement: Supplementary file 1 — Supplementary Information [file 41467_2024_46322_MOESM1_ESM.pdf]

Supplemental Figure 1

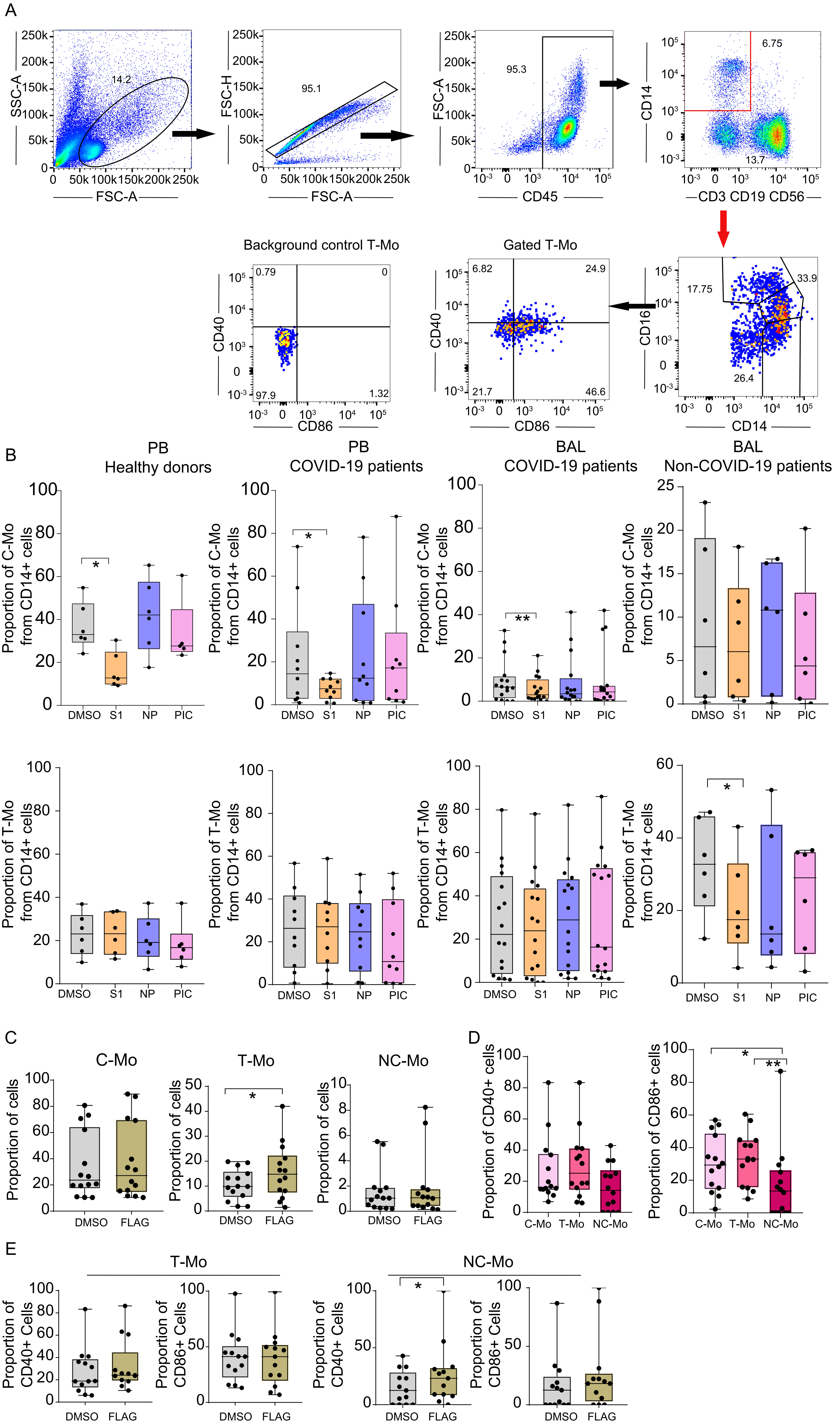

Supplemental Figure 1. Analysis of changes in Mo subsets from PBMC after exposure to SARS-CoV-2 proteins and TLR ligands. (A) Representative flow cytometry gating strategy defining CD14+ CD3-CD19-CD56- Mo from PBMC and identifying Classical (C-Mo), Transitional (T-Mo) and non-classical (NC-Mo) Mo subsets based on differential levels of CD16 and CD14. Levels of CD40 vs CD86 on gated cells from each population were also analyzed. A representative flow cytometry plot on gated T-Mo and the background control for this subset is also shown. (B): Proportions of C-Mo and T-Mo from cultures of bulk PBMC (n=10) and BAL (n=16) from COVID-19 patients, PBMC from healthy donors (n=6) or BAL from non-COVID-19 patients (n=6) in DMSO (grey), S1 peptide (orange), NP peptide (purple) and Poly I:C (PIC, pink) condition. (C): Proportions of C-Mo, T-Mo and NC-Mo in pre-isolated CD14+ Mo (C) after 16h of culture in the presence of DMSO (grey) and flagellin (FLAG, khaki) (n=14). (D) Proportions of CD40+ and CD86+ cells in C-Mo (light pink), T-Mo (pink) and NC-Mo (magenta) populations at baseline conditions in DMSO (n=14). (E): Percentages of CD40+ and CD86+ cells in the indicated Mo subsets on each of the mentioned culture conditions in (C) n=13. Statistical significance was calculated using a two tailed Wilcoxon test: *p<0.05, **p<0.01. Data are represented as box and whiskers with bars representing maximum and minimum values and with median highlighted as a line.

Supplemental Figure 2

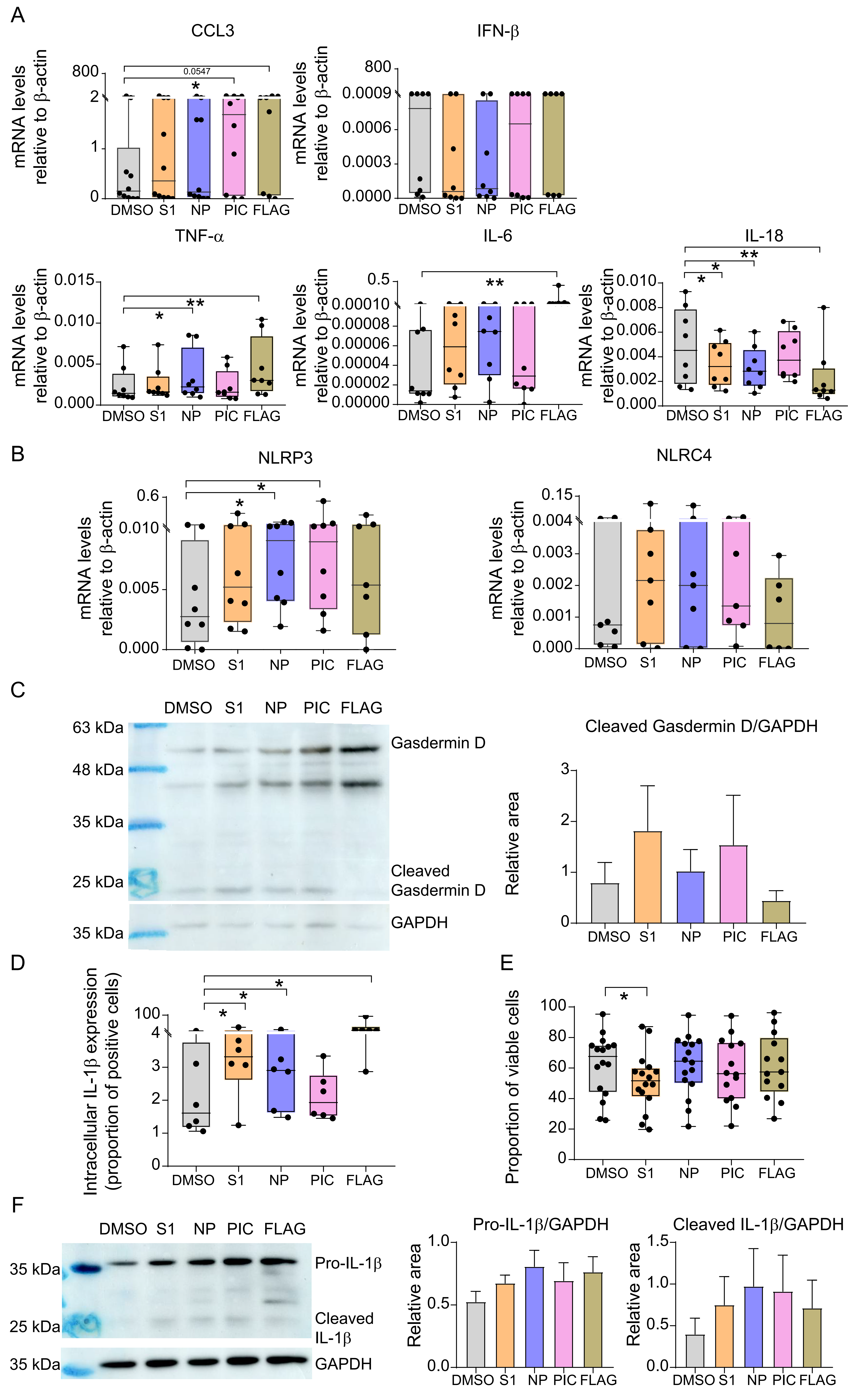

Supplemental Figure 2. Transcriptional levels of proinflammatory cytokines and inflammasome sensors in Mo exposed to SARS-CoV-2 proteins and TLR ligands. (A, B): mRNA levels quantified by RT- qPCR of CCL3 (n=10), IFNβ, TNF-α, IL-6 and IL-18 (n=8) (A) and NLRP3 (n=8) and NLRC4 (n=7) inflammasome sensors (B) in isolated CD14+ Mo cultured in the presence of DMSO or S1 peptide (orange), NP peptide (purple), Poly I:C (PIC, pink), flagellin (FLAG, khaki). Transcriptional levels were normalized to the β-actin expression. (C-F): Representative Western blot analysis of Gasdermin D (C) and IL-1β (F) uncleaved and processed isoforms and GAPDH in Mo cultured in the mentioned conditions. Quantification of results relative to GAPDH from three independent experiments for both proteins is shown on the right. Data in these panels is represented as mean and error bars correspond to SEM. (D, E): Proportions of IL-1β+ (D; n=6) and viable (E; n=16) Mo in the previously mentioned culture conditions. Statistical significance in (A, B, D, E) was calculated using a two tailed Wilcoxon test: *$p<0.05$, **$p<0.01$. Data are represented as box and whiskers with bars representing maximum and minimum values and with median highlighted as a line.

Supplemental Figure 3

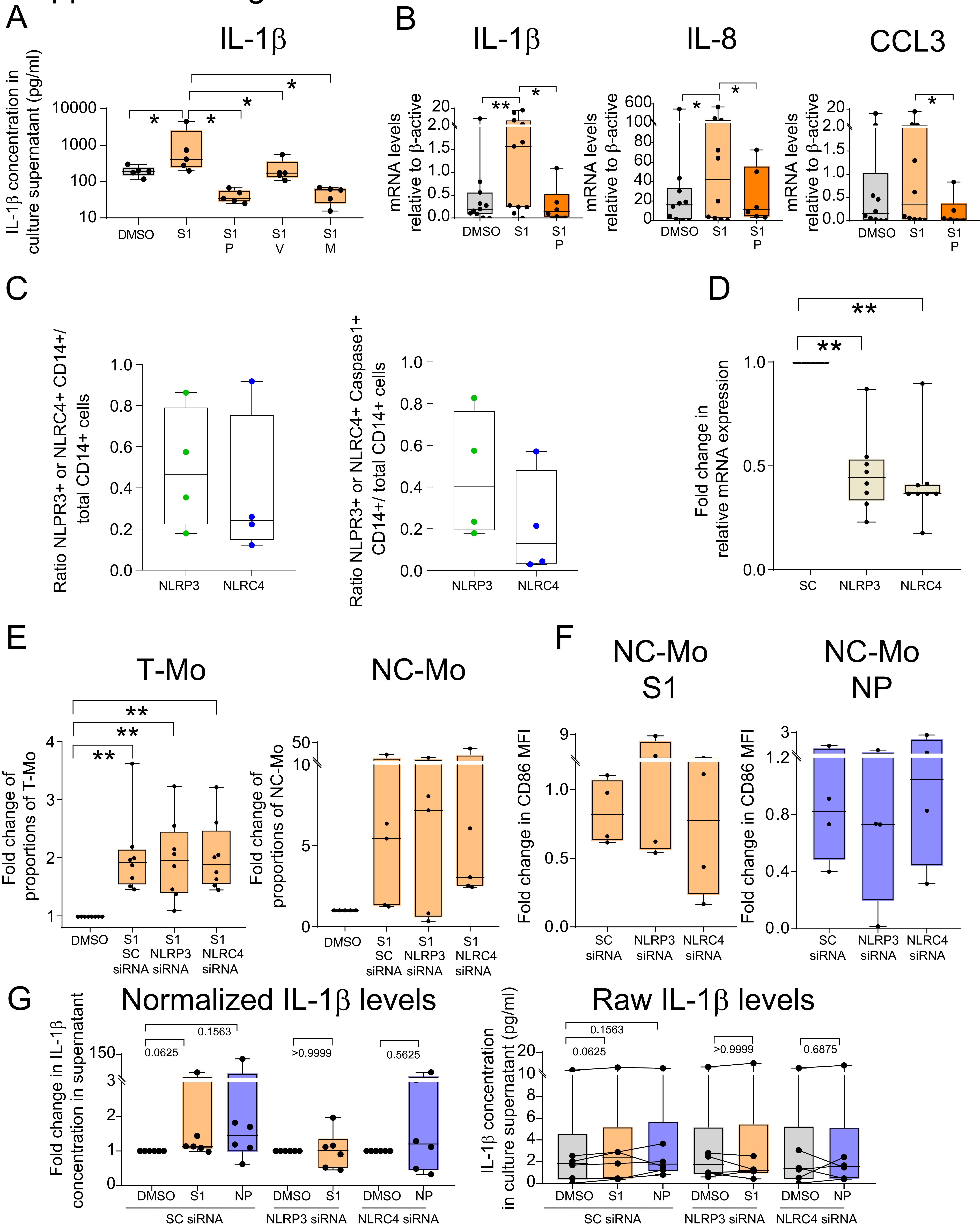

Supplemental Figure 3. Pharmacological and siRNA mediated inhibition of the inflammasome and NFκB. (A): ELISA quantification of the concentrations of secreted IL-1β protein in culture supernatants of Mo cultured in the presence of DMSO (grey) versus S1 (orange) in the presence of DMSO or Parthenolide (P), Z-VAD-FMK (V), and MCC950 (M) (n=5). Statistical significance was calculated using a one-tailed Wilcoxon test *p<0.05. (B) RT-qPCR analysis of IL-1β (n=11), IL-8 (n=10) and CCL3 (n=10) in Mo cultured in the absence or the presence of DMSO (grey), S1with DMSO (orange) or with S1 and Parthenolide (P) (dark orange). (C): Quantification of ratios of cells co-expressing NLRP3 (green) or NLRC4 (blue) with CD14 alone (left) or with caspase 1 (right) from total CD14+ cells in lung tissue from n=3 critical COVID-19 patients. (D): RT-qPCR quantification of NLRP3 and NLRC4 in cells treated with specific siRNAs relative to cells nucleofected with Scramble (SC) siRNAs (n=8). Data are normalized to levels of mRNA in control condition (Scramble) and relative to β-actin expression. (E): Fold change in proportions of T-Mo (n=8) and NC-Mo (n=5) subsets from Mo nucleofected with scramble (SC) siRNA or siRNA specific for NLRP3 and NLRC4 and treated with S1 (orange) compared to DMSO control conditions. (F): Fold change in CD86 MFI on NC-Mo in response to viral proteins (S1 (orange) or NP (purple)) after nucleofection with scramble (SC) or NLRP3 and NLRC4-specific siRNAs (n=4). (G): Impact of nucleofection of Mo with scramble (SC), NLRP3 or NLRC4 specific-siRNAs in levels of secreted IL-1β in supernatants after exposure to S1 and NP compared to DMSO conditions. Normalized and raw data are shown in left and right plots, respectively. Statistical significance of differences for (B, C, D) were calculated using a two-tailed Wilcoxon test *p<0.05, **p<0.01. Data are represented as box and whiskers with bars representing maximum and minimum values and with median highlighted as a line

Supplemental Figure 4

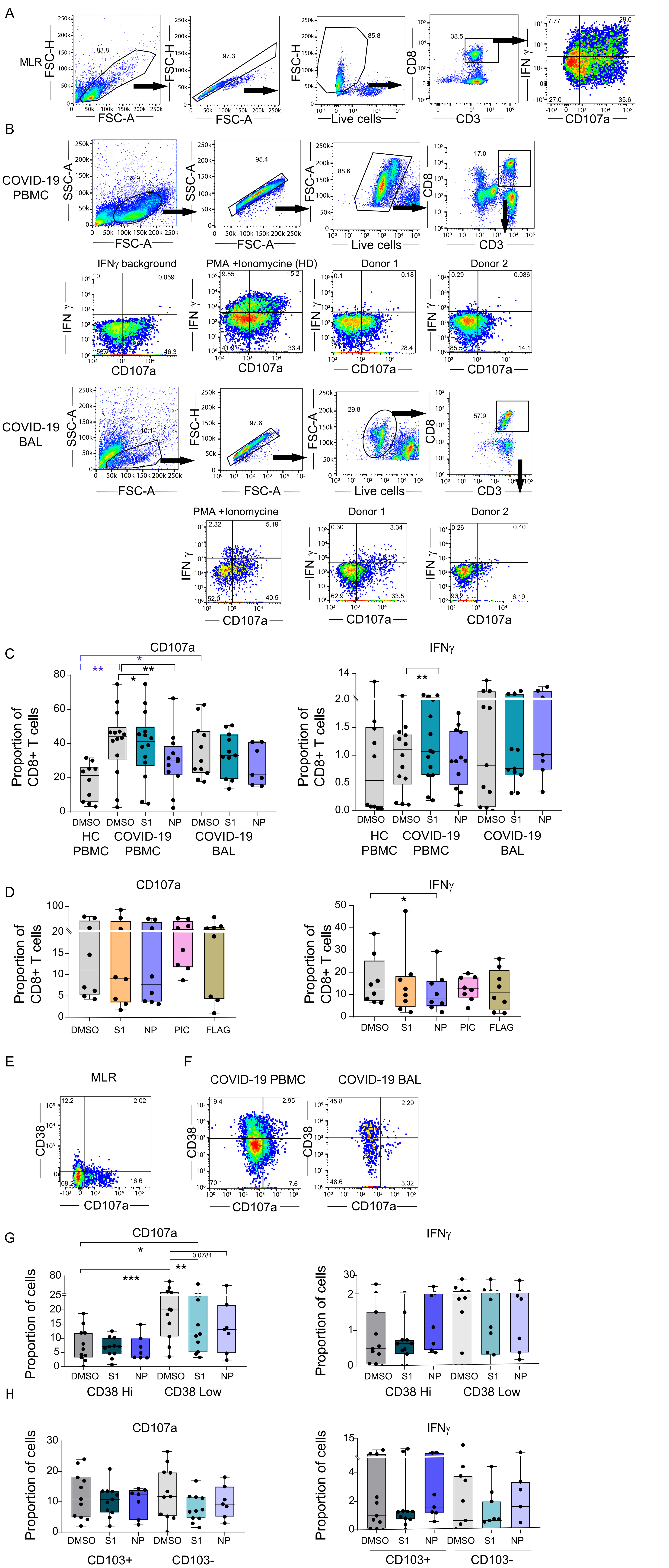

Supplemental Figure 4. CD8+ T cell responses induced by primed Mo and specifically induced in COVID-19 PBMC and BAL after stimulation with SARS-CoV-2 S1 and NP proteins. (A, B): Representative flow cytometry gating strategy defining CD8+ T cells and expression of IFNγ and CD107a in MLR assays (A) and in PBMC and BAL from COVID-19 patients (B). Positive controls with PMA and Ionomycin are shown, as well as representative examples from selected patients. (C): Expression of CD107a (left) and IFNγ (right) on CD8+ T cells from PBMC (n=14) and from COVID-19 BAL samples (n=11) from critical COVID-19 patients at baseline and after stimulation with S1 (green) and NP (purple) peptides or DMSO (grey). Baseline levels from PBMCs obtained from healthy donors cultured in DMSO (grey) are included for comparison purposes (n=10). (D): Proportions CD107a+ and IFNγ+ CD8+ T cells in MLR assays performed in the presence of allogeneic Mo cultured in the presence of DMSO (grey) or S1 (orange), NP (purple), Poly I:C (PIC; pink), Flagellin (FLAG, khaki) and corresponding to normalized data shown in figure 4 (n=8). (E, F): Representative flow cytometry dot plots showing CD38 vs CD107a expression in CD8+ T cells from MLR assays (E) and from COVID-19 BAL and PBMC samples (F). (G, H): Proportions of IFNγ+ and CD107a+ cells in CD38Hi and CD38Low (G) or in CD103+ and CD103- (H) CD8+ T cells from BAL exposed to DMSO (dark grey, grey) o SARS-CoV-2 S1 (dark green, green) or NP (blue, light purple) (n=11). A Mann-Whitney test was used to statistical differences between samples from different cohorts. Statistical differences between different experimental conditions in the same groups were calculated using a two-tailed Wilcoxon matched pairs test *p<0.05, **p<0.01, ***p<0.001. Data are represented as box and whiskers with bars representing maximum and minimum values and with median highlighted as a line.

Supplemental Figure 5

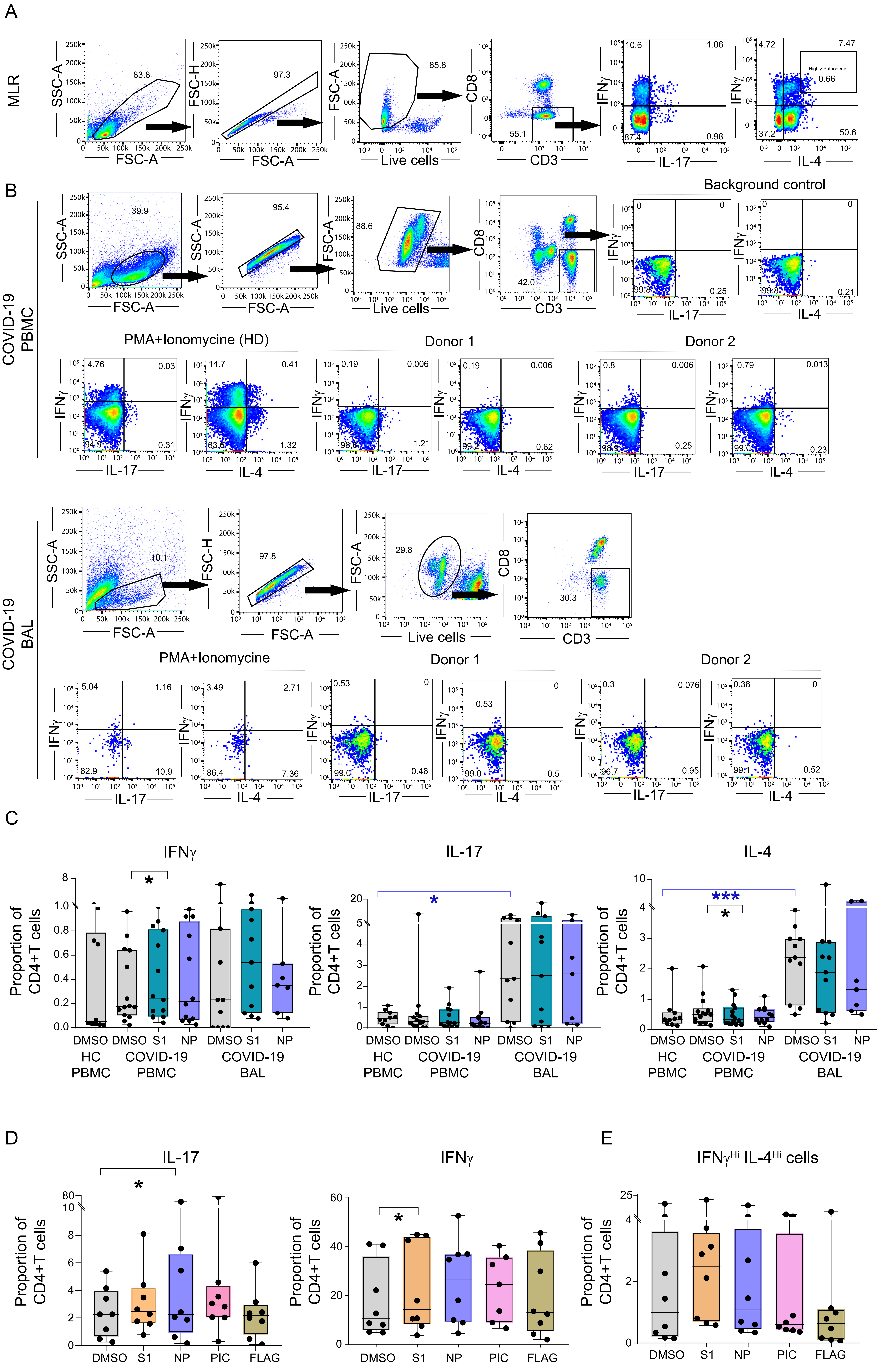

Supplemental Figure 5. CD4+ T cell responses induced by primed Mo and specifically induced in COVID-19 PBMC and BAL after stimulation with SARS-CoV-2 S1 and NP proteins. (A, B): Representative flow cytometry gating strategy defining CD4+ T cells and expression IFNγ, IL-17 and IL-4 in MLR assays (A) and in PBMC and BAL from COVID-19 patients (B). Positive controls with PMA and Ionomycin are shown as well as representative examples from selected patients. Background controls are also included (C): Expression of IFNγ, IL-17 and IL-4 on CD4+ T cells from PBMC of healthy donors (n=10) and COVID-19 (n=14) and from COVID-19 BAL (n=11) samples at baseline and after stimulation with S1 (green) and NP (purple) peptides. Baseline levels from PBMCs obtained from healthy donors cultured in DMSO (grey) are included for comparison purposes. (D, E): Proportions of IL17+, IFNγ+ (D) and cells co-expressing high levels of IFNγ and IL-4 (E) in CD4+ T cells from MLR assays performed with Mo exposed to DMSO (grey) or S1 (orange), NP (purple), Poly I:C (PIC; pink), Flagellin (FLAG, khaki) (n=8). Mann-Whitney test was used to compare samples from different study cohorts. Statistical significances between different experimental conditions within the same groups were calculated using a two-tailed Wilcoxon matched pairs test *$p<0.05$, ***$p<0.001$. Data are represented as box and whiskers with bars representing maximum and minimum values and with median highlighted as a line.

Supplemental figure 6

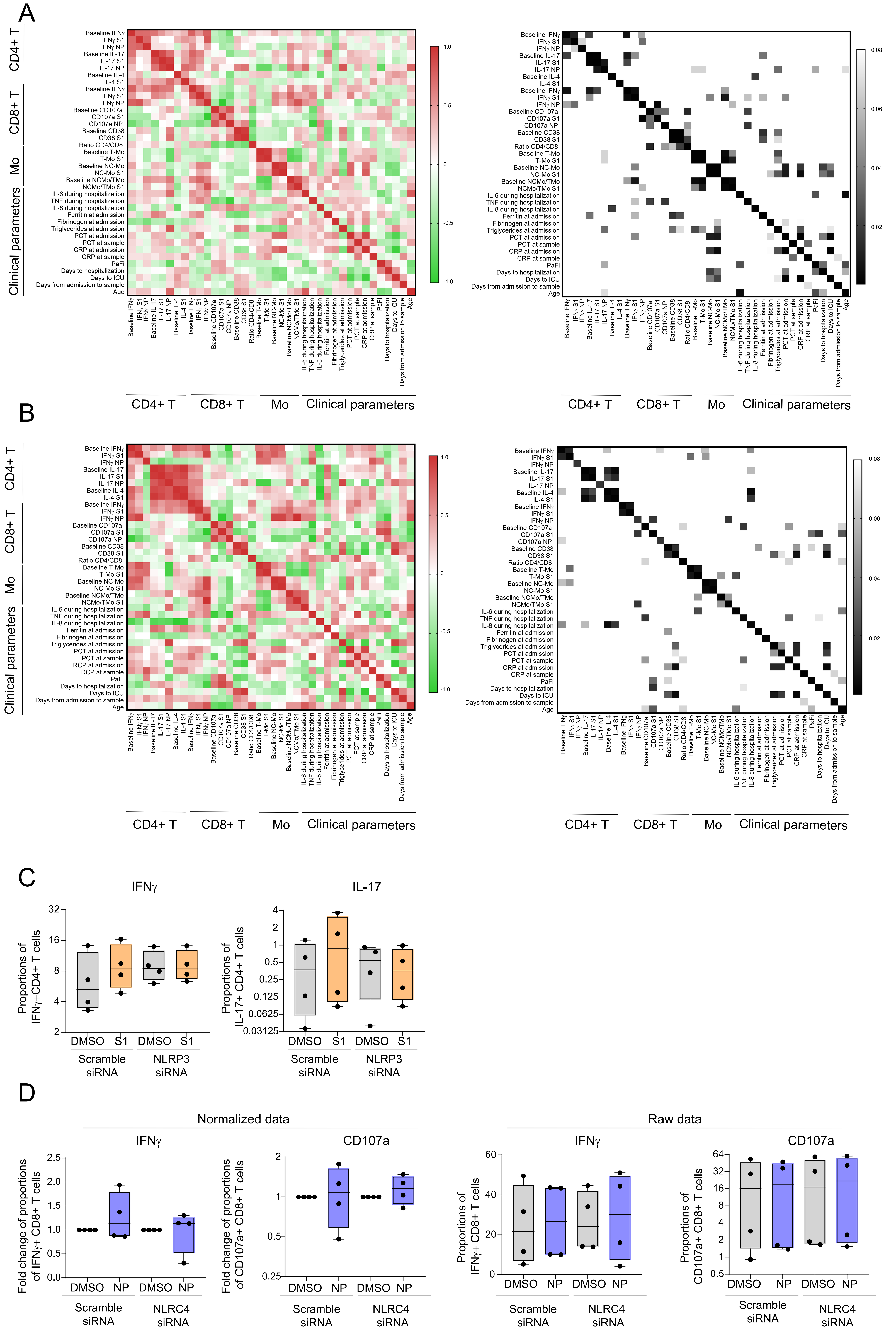

Supplemental Figure 6. Correlation networks between Mo, T cell and clinical patterns in BAL from critical COVID-19 patients. (A, B): Heatmaps showing spearman correlation networks of the indicated Mo subsets, CD4+ and CD8+ T cell responses present at baseline or after SARS-CoV-2 peptide stimulation in all BAL samples from COVID-19 patients tested (A) n=12 or exclusively those in which IL-17 induction was observed in response to SARS-CoV-2 S1 or NP peptide stimulation (B) n=7. Heatmaps of the left show colors representing positive (red) or negative (green) R values of association. Heatmaps on the right show levels of statistical significance for the same corresponding networks. (C, D) Proportions of IFNγ+ (left, C and D) and IL-17+ (C) or CD107a+ (D) (right) CD4+ and CD8+ T cells after culture with allogeneic Mo treated with either Scramble, NLRP3 or NLRC4 specific siRNAs and in the absence (DMSO) or presence of S1 (orange) or NP (purple) stimulation (n=4). Data are represented as box and whiskers with bars representing maximum and minimum values and with median highlighted as a line.

**Number of PB samples**	n=17
**Age *median (min-max)***	69 (44-78)
**Sex male *n (%)***	12 (71)
**Days since symptoms to admission *median (min-max)***	4.5 (0-25)
**Days since admission to sample *median (min-max)***	26 (5-47)
**Days since admission to ICU *median (min-max)***	6 (0-19)
**Admission to ICU *n (%)***	17 (100)
**Comorbidities *n (%)***	
Arterial hypertension	9 (53)
Diabetes mellitus	5 (29)
Dyslipidemia	7 (41)
Lung disease	1 (6)
**Analytics parameters *median (min-max)***	
$PaFiO_2$ ($PaO2/FiO2$)	146 (60-367)
**PCT at sample collection (ng/ml)**	0.3 (0.06-2.87)
**PCT at admission (ng/ml)**	0.16 (0.07-1)
Lymphocytes miles/mm^3	850 (420-5450)
LDH (U/L)	288 (145-548)
D-DIMER (μg/ml)	2.2 (1.33-14.7)
**CRP at sample collection (mg/dl)**	9 (0.13-20.8)
CRP at admission (mg/dl)	7.3 (3.4-24)
GGT (U/l)	201.5 (25-473)
Glucose (mg/dl)	152 (87-207)
Ferritin at admission (ng/ml)	712 (114-2733)
Triglycerides at admission (mg/dl)	271 (43-674)
Fibrinogen at admission (mg/dl)	641 (389-1152)
IL-6 (pg/ml)	31 (2-56)
**Exitus *n (%)***	7 (41)
**Superinfection *n (%)***	
Bacterial	8 (47)
Fungal	7 (41)
Both	5 (29)
**Treatment *n (%)***	
Antibiotics	11 (65)
Glucocorticoids	13 (76)
Biologics	6 (35)
Antiviral	0 (0)

Supplemental table 1. Peripheral blood samples from COVID-19 patients used in the study.

**Number of Samples**	N=17
**Age *median (min-max)***	69 (44-78)
**Sex male *n (%)***	12 (71)
**Days since symptoms to admission *median (min-max)***	4.5 (0-25)
**Days since admission to sample *median (min-max)***	26 (5-47)
**Days since admission to ICU *median (min-max)***	6 (0-19)
**Admission to ICU *n (%)***	17 (100)
**Comorbidities *n (%)***	
Arterial hypertension	10 (59)
Diabetes mellitus	6 (35)
Dyslipidemia	11 (65)
Lung disease	1 (6)
**Analytics parameters *median (min-max)***	
$PaFiO_2$ (PaO2/FiO2)	158 (94.3-211.3)
**PCT at sample collection (ng/ml)**	0.3 (0.09-1.68)
**PCT at admission (ng/ml)**	0.13 (0.04-1.01)
Lymphocytes miles/mm^3	780 (50-5450)
LDH (U/L)	284 (145-426)
D-DIMER (µg/ml)	2.07 (1.09-14.7)
**CRP at sample collection (mg/dl)**	9.6 (0.81-20.78)
CRP at admission (mg/dl)	7.3 (3.4-24)
GGT (U/l)	131 (25-473)
Glucose (mg/dl)	153 (92-207)
Ferritin at admission (ng/ml)	934 (313-2733)
Triglycerides at admission (mg/dl)	137 (43-451)
Fibrinogen at admission (mg/dl)	688 (540-952)
IL-6 (pg/ml)	27.65 (2-56)
**Exitus *n (%)***	7 (41)
**Superinfection *n (%)***	
Bacterial	11 (65)
Fungal	7 (41)
Both	6 (35)
**Treatment *n (%)***	
Antibiotics	12 (71)
Glucocorticoids	17 (100)
Biologics	6 (35)
Antiviral	1 (6)

Supplemental table 2. Bronchoalveolar lavage samples from COVID-19 patients used in the study.

Number of Non-COVID-19 BAL samples	n=6
**Age median (min-max)**	69 (38-77)
**Pathologies**	
**Cancer n (%)**	3 (50)
**Intersticial lung disease (ILD) n (%)**	3 (50)

Supplemental table 3. Parameters of Non-COVID-19 patients whose BAL samples were used in the study

Antibody	Fluorochrome	Provider	Dilution	Clone	Ref.
**CD14**	PE	BioLegend	1:50	63D3	367104
**CD14**	APC-Cy7	BD	1:50	MφP9	557831
**CD16**	PB	BioLegend	1:50	3G8	302032
**CD40**	FITC	BioLegend	1:50	5C3	334306
**CD86**	PeCy7	BioLegend	1:50	BU63	374210
**CD3**	PB	Immunostep	1:50	33-2A3	3CFB1-100T
**CD107a**	APC	BioLegend	1:300	H4A3	328620
**CD107a**	BV510	BioLegend	1:200	H4A3	328631
**CD38**	PeCy7	BioLegend	1:50	HB-7	356608
**CD45**	V500	BD	1:50	HI30	560777
**CD1c**	PE	BD	1:50	F10/21A3	564900
**CD19**	PercP/Cyanine5.5	BioLegend	1:50	SJ25C1	363016
**CD56**	PercP/Cyanine5.5	BioLegend	1:50	5.1H11	362506
**CD3**	PercP/Cyanine5.5	BioLegend	1:50	HIT3a	300328
**IL-1β**	FITC	BioLegend	1:100	H1b-98	511705
**HLA-DR**	APC	BioLegend	1:50	3D12	342606
**IL-17**	PE	BioLegend	1:100	BL168	512306
**IL-17**	APC	Invitrogen	1:100	eBio64DEC17	17-7179-42
**IL-4**	PE	BD	1:30	340451	340451
**IL-4**	APC	BD	1:100	MP4-25D2	554486
**CXCR5**	PE	BioLegend	1:50	42D1	338705
**CD8**	PercP	BioLegend	1:50	HIT8a	300922
**IFNγ**	BV605	BioLegend	1:50	B27	506542
**IFNγ**	FITC	BD	1:100	4S.B3	554551
**CD103**	BV711	BioLegend	1:50	Ber-ACT8	350222

Supplemental table 4. List of antibodies used for Flow cytometry analysis in the study.

Specifitiy	Species Origin	Reactivity	Provider	Clone	Ref.
**NLRC4**	Rabbit	Human	abcam	Polyclonal	ab115537
**IFNγ**	Rabbit	Human	abcam	EPR21704	ab231036
**CD14**	Mouse	Human	abcam	4B4F12	ab182032
**NLRP3**	Rabbit	Human	Cell Siganling	D4D8T	15101
**IL-17**	Goat	Human	RyD Systems	Polyclonal	AF-317-SP
**Caspase-1**	Goat	Human	RyD Systems	Polyclonal	AF6215
**CD3**	Rabit	Human	Dako	F7.2.38	M7254
**IgG**	Donkey	Rabbit	Invitrogen	Polyclonal	A-21206
**IgG**	Donkey	Goat	Invitrogen	Polyclonal	A-11057
**IgG**	Donkey	Mouse	Invitrogen	Polyclonal	A-31571

Supplemental Table 5. List of antibodies used for Immunohistochemistry in the study.

	Survivors (n=6)	Exitus (n=6)	*p*-value
**NC Mo at baseline**	17.2 (11.1-22.4)	7.8 (1.8-9.4)	0.05
**NC Mo after S1**	30.3 (9.3-30.6)	9.8 (3.8-15.4)	0.08
**NC Mo after NP**	20.5 (12.8-25.6)	5.5 (3.2-9.6)	0.02
**IL-4 at baseline**	0.8 (0.7-2.4)	2.9 (2.3-3.4)	0.04

NC Mo: non-classical monocytes. NP: nucleoprotein.

Data are shown as median (interquartile range). Non-parametric Mann-Whitney tests were used to assess statistical significance. p<0.05 was considered as statistically significant.

	No superinfection (n=5)	Bacterial superinfection (n=3)	Bacterial and fungal superinfection (n=4)	*p*-value	Post-hoc Dunn's test
**CD4 IL-17 at baseline**	2.4 (2.3-6.4)	0.2 (0-1.4)	7.1 (5.1-9)	0.03	Bacterial and fungal>bacterial
**CD4 IL-17 after S1**	4.1 (2.5-4.4)	0 (0-0.1)	10.1 (5-14.6)	0.02	Bacterial and fungal>bacterial; no superinfection> bacterial
**CD4 IL-17 after NP**	2.6 (0.2-3.4)	0.3 (0.2-0.5)	8.2 (6.3-10)	0.1	____________

NP: nucleoprotein.

Data are shown as median (interquartile range). Non-parametric Kruskal-Wallis tests followed by post-hoc Dunn's tests were used to assess statistical significance. p<0.05 was considered as statistically significant.

Supplemental Table 6. Statistical analysis of COVID-19 patients distribution based on survival and bacterial or fungal superinfection.

Molecular weight control: BlueStar Plus Prestained Protein Marker (Nippon Genetics)

Chemiluminiscence+Marker

Chemiluminiscence

Phosphorylated p65, total p65 and GAPDH included in Main figure 2B analysis

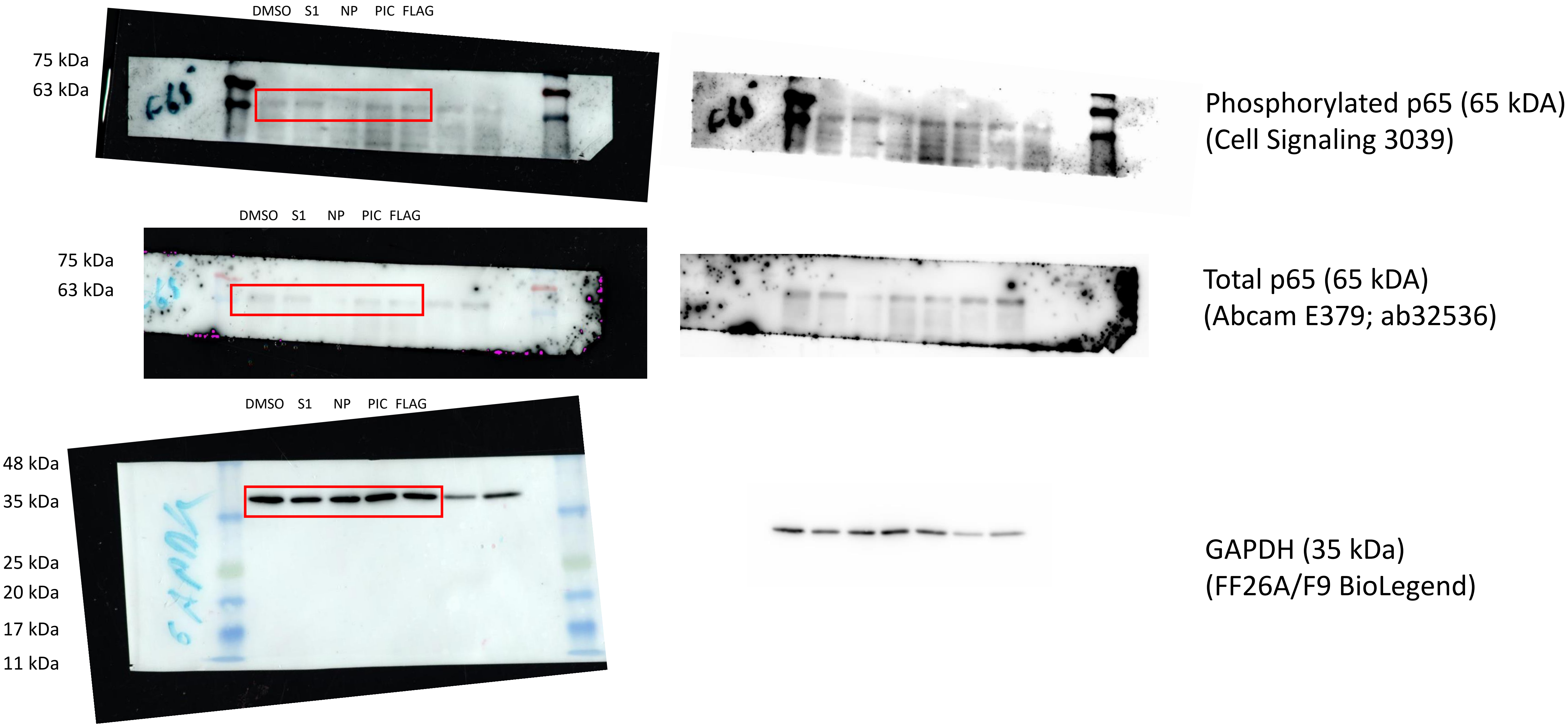

Chemiluminiscence+Marker

Chemiluminiscence

Phosphorylated p65, total p65 and GAPDH included in Main figure 2B analysis

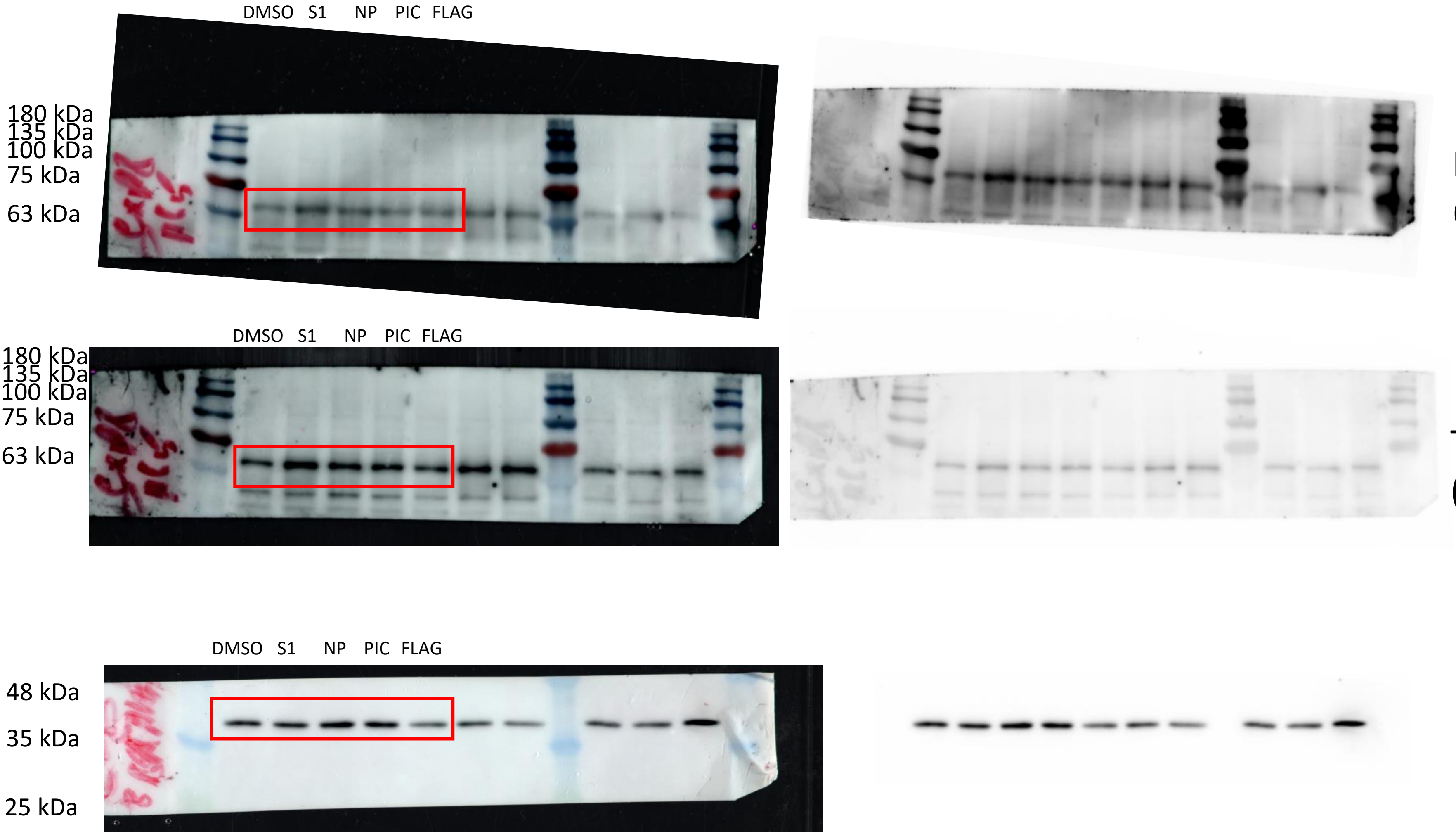

Phosphorylated p65 (65 kDA)
(Cell Signaling 3039)

Total p65 (65 kDA)
(Abcam E379; ab32536)

GAPDH (35 kDa)
(FF26A/F9 BioLegend)

Chemiluminiscence+Marker

Chemiluminiscence

Phosphorylated p65, total p65 and GAPDH included in Main figure 2B analysis

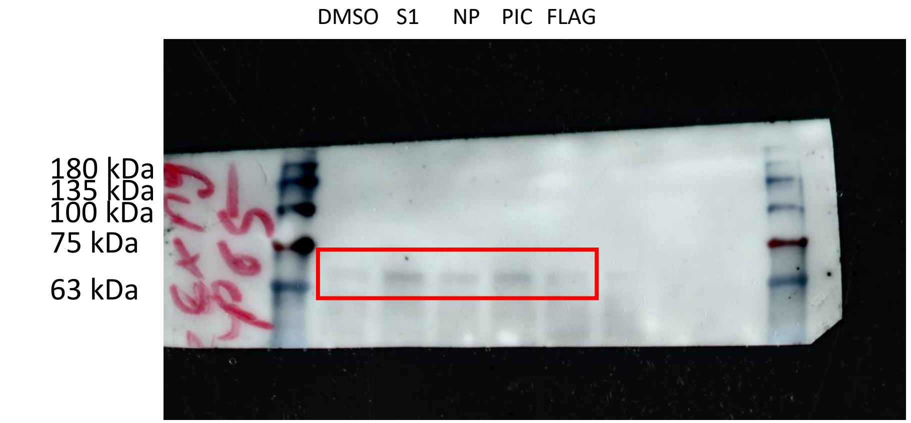

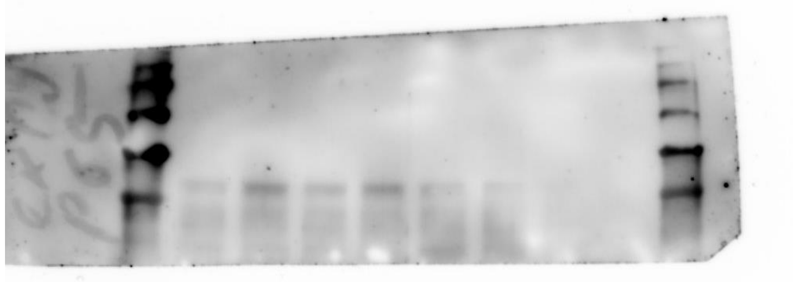

Phosphorylated p65 (65 kDA)
(Cell Signaling 3039)

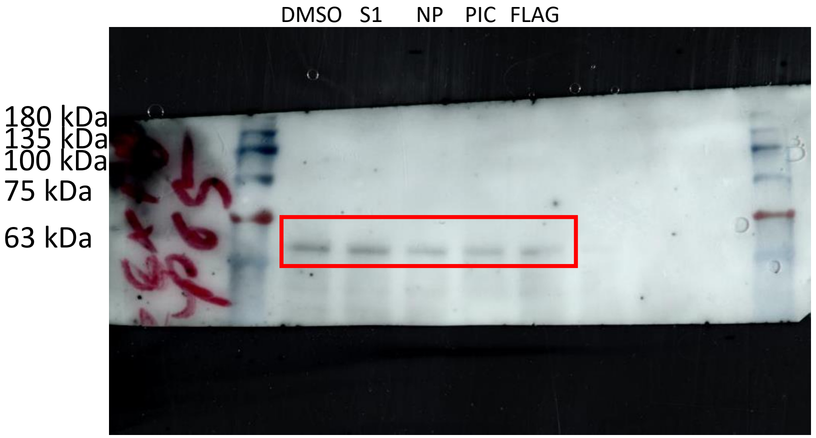

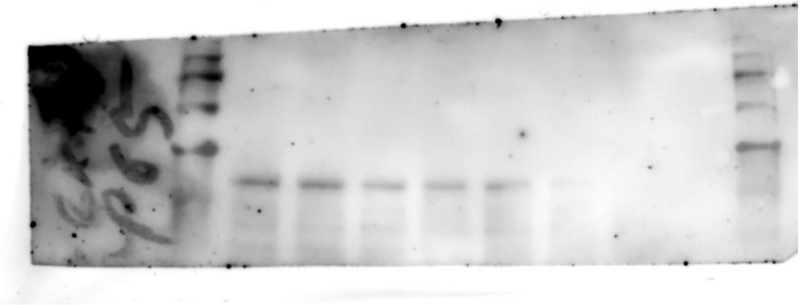

Total p65 (65 kDA)
(Abcam E379; ab32536)

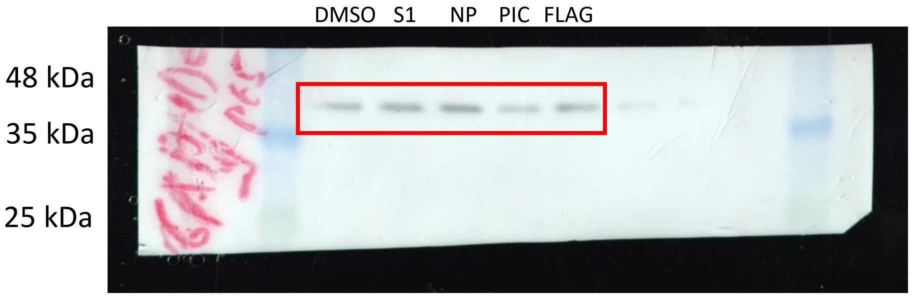

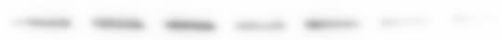

GAPDH (35 kDa)
(FF26A/F9 BioLegend)

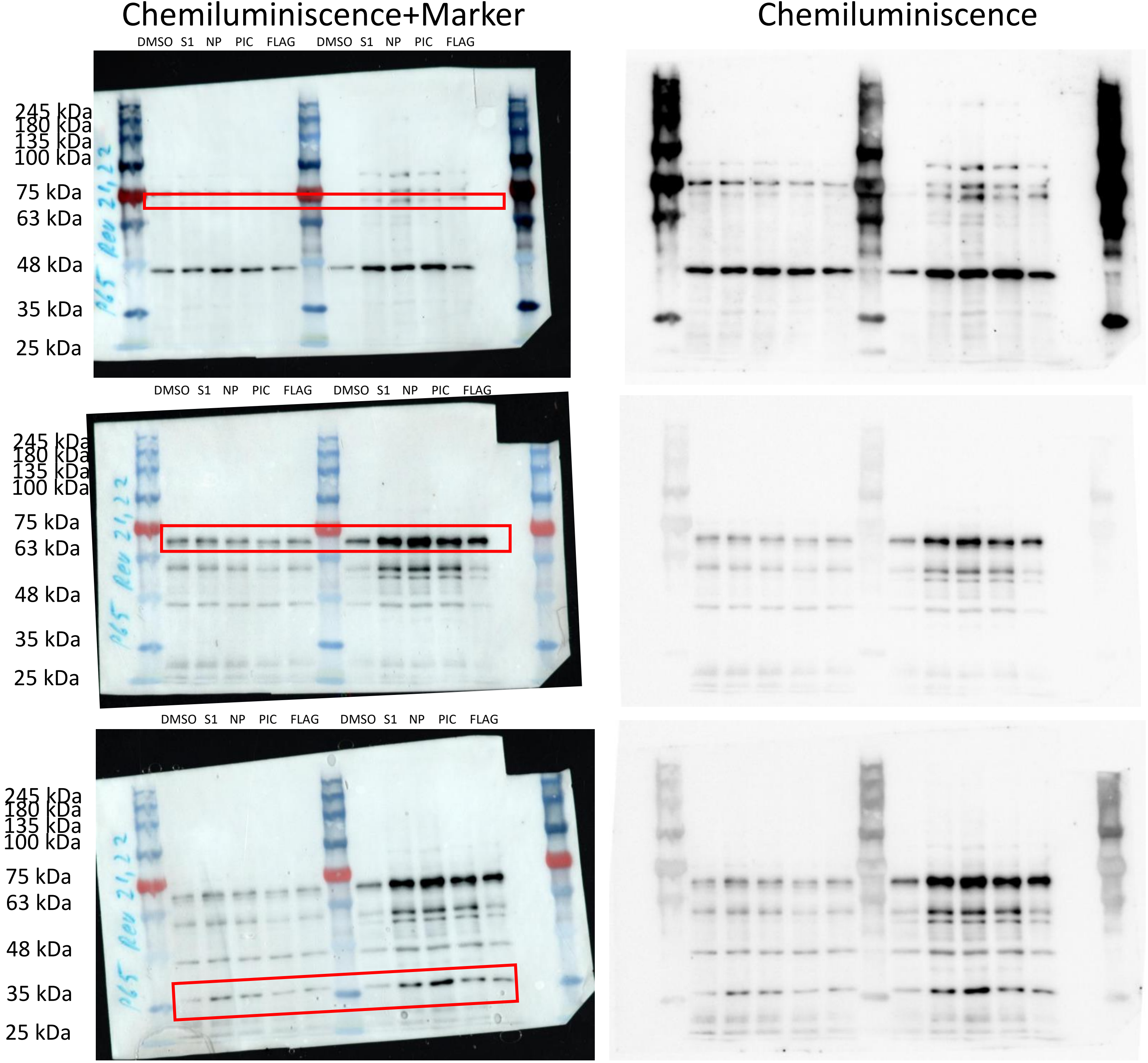

Phosphorylated p65, total p65 and GAPDH included in Main figure 2B analysis

Phosphorylated p65 (65 kDA)
(Cell Signaling 3039)

Total p65 (65 kDA)
(Abcam E379; ab32536)

GAPDH (35 kDa)
(FF26A/F9 BioLegend)

Chemiluminiscence+Marker

Chemiluminiscence

Caspase-1 and GAPDH included in Main figure 2C analysis

S1 DMSO

180 kDa
135 kDa
100 kDa
75 kDa
63 kDa
48 kDa

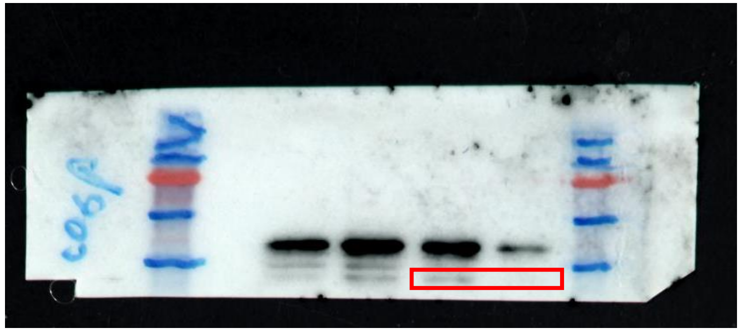

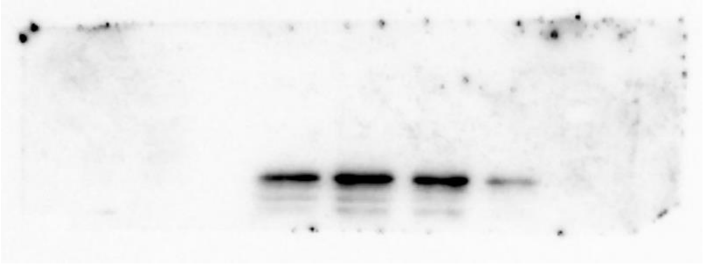

Cleaved Caspase-1 (52 kDa) (AF6215; R&D Systems)

S1 DMSO

35 kDa
25 kDa
20 kDa
17 kDa
11 kDa

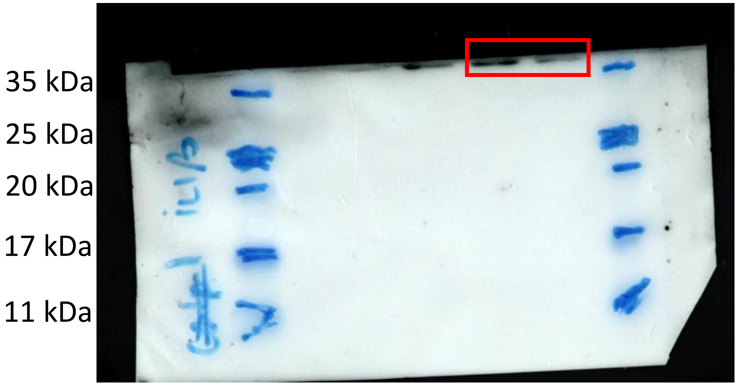

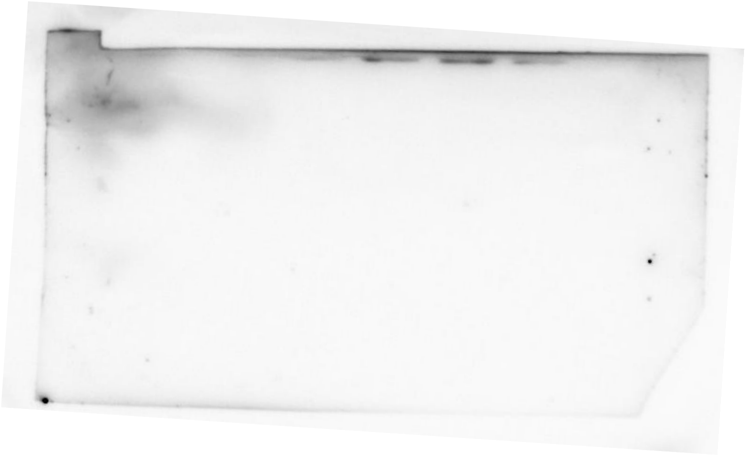

GAPDH (35 kDa) (FF26A/F9 BioLegend)

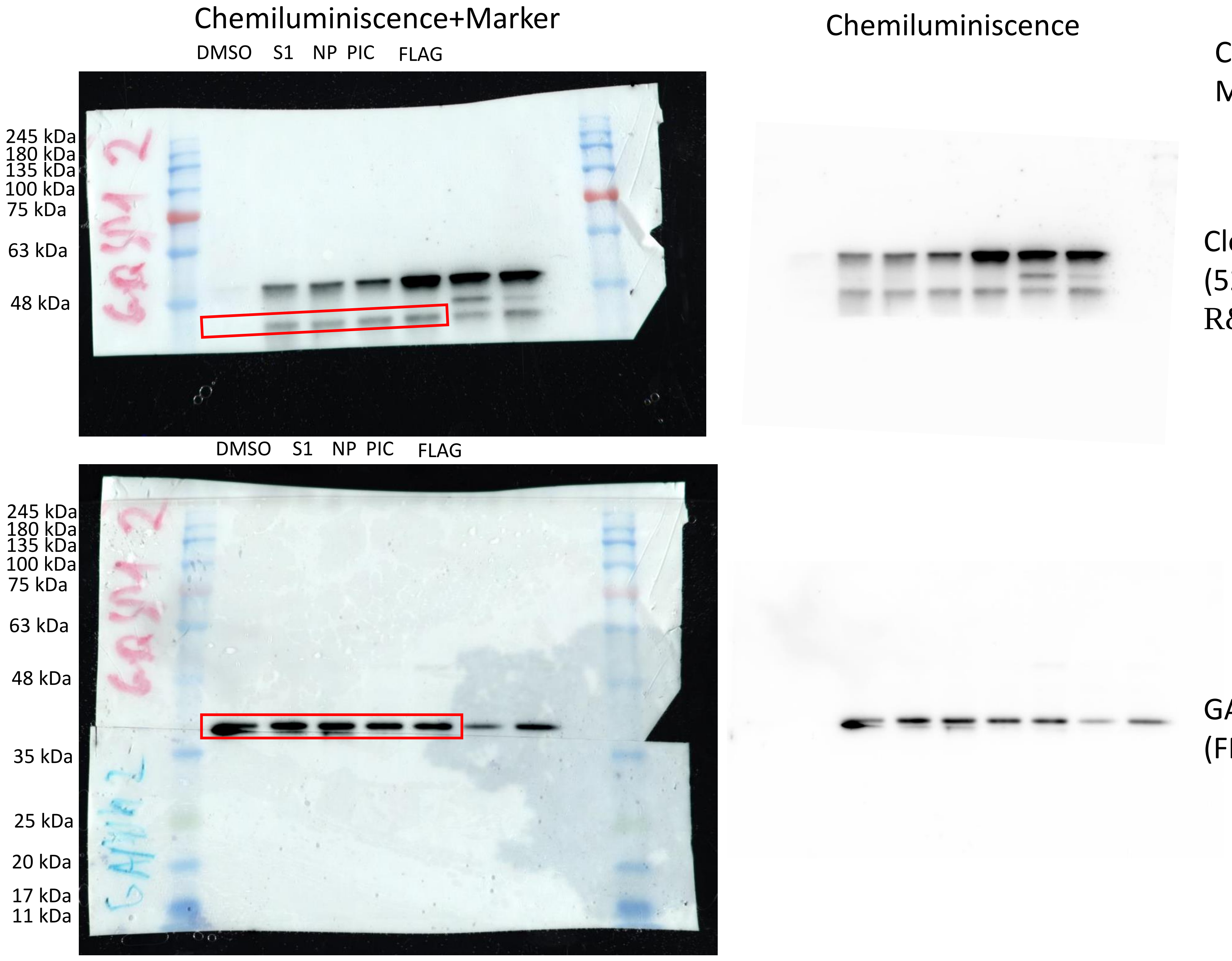

Caspase-1 and GAPDH included in Main figure 2C analysis

Cleaved Caspase-1 (52 kDa) (AF6215; R&D Systems)

GAPDH (35 kDa) (FF26A/F9 BioLegend)

Chemiluminiscence+Marker

Chemiluminiscence

Caspase-1 and GAPDH included in Main figure 2C analysis

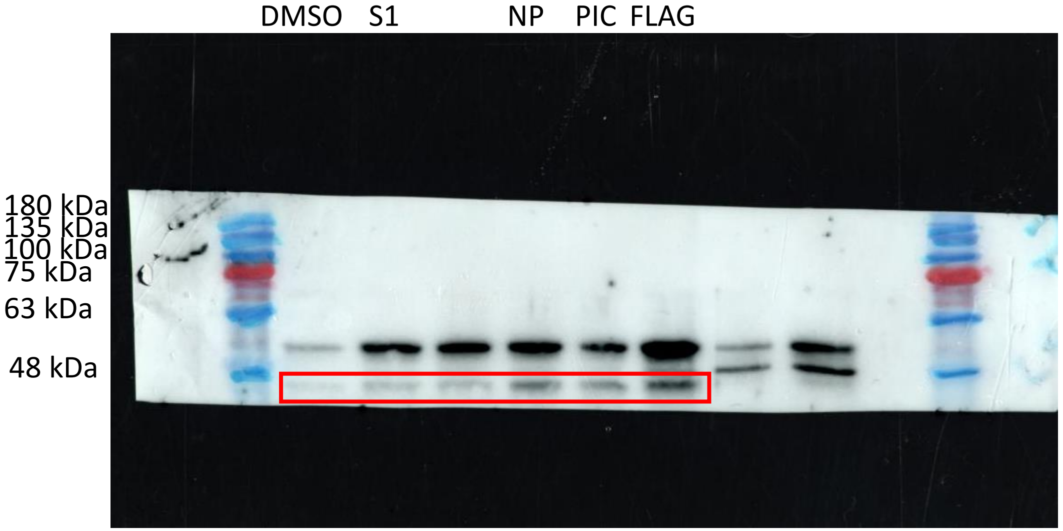

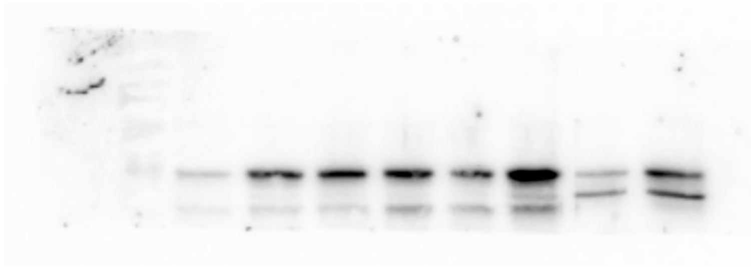

Cleaved Caspase-1 (52 kDa) (AF6215; R&D Systems)

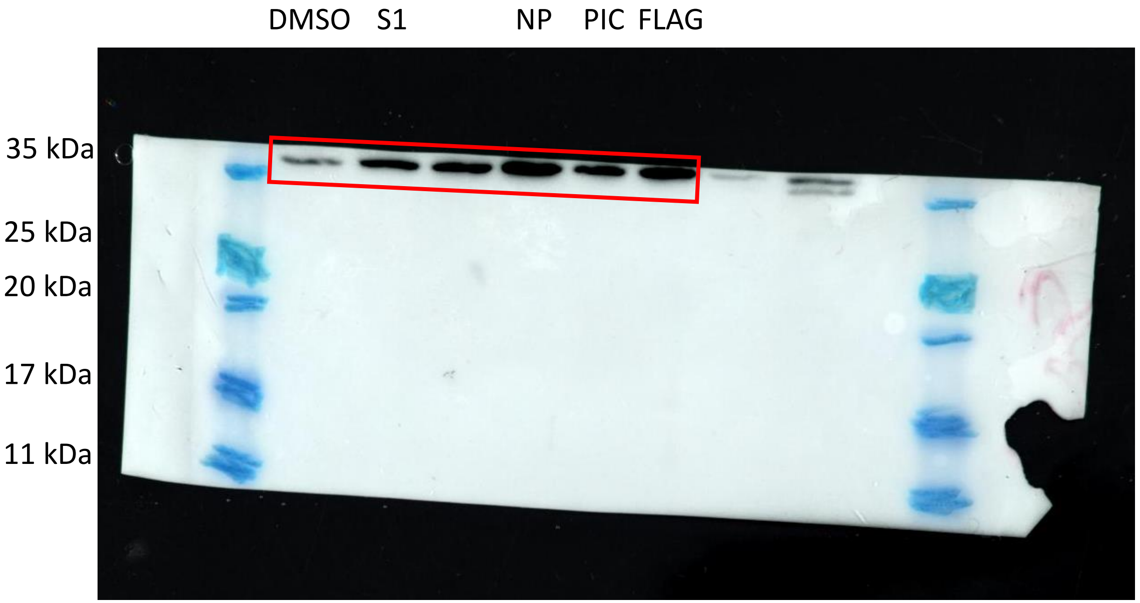

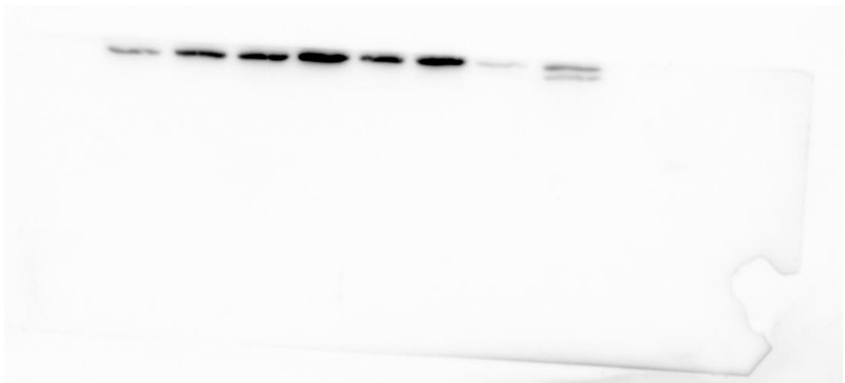

GAPDH (35 kDa) (FF26A/F9 BioLegend)

Chemiluminiscence+Marker

Chemiluminiscence

Caspase-1 and GAPDH included in Main figure 2C analysis

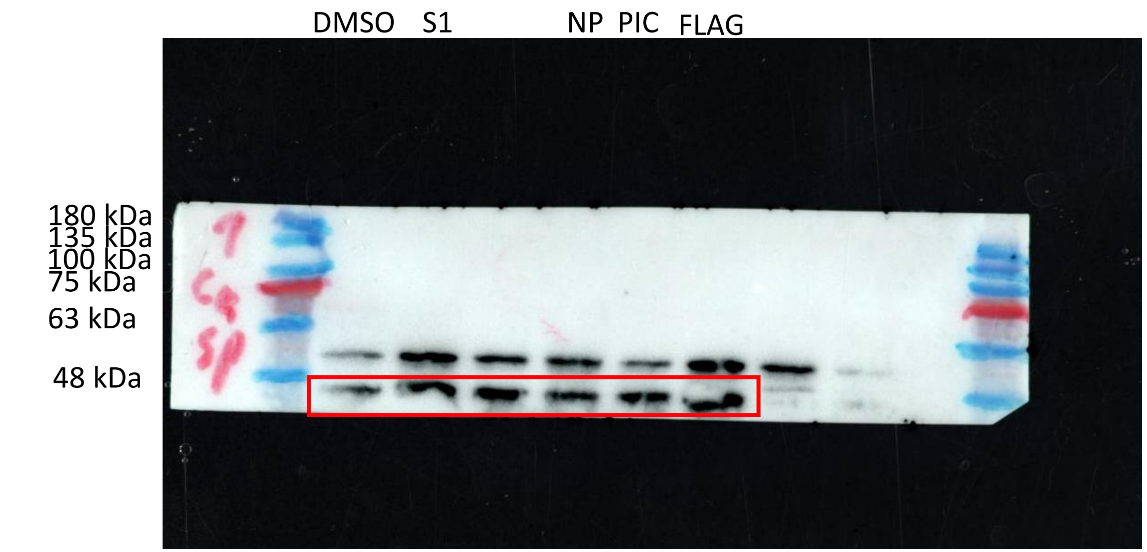

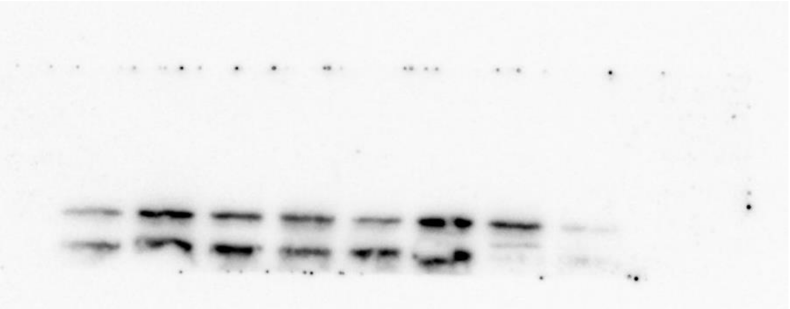

Cleaved Caspase-1 (52 kDa) (AF6215; R&D Systems)

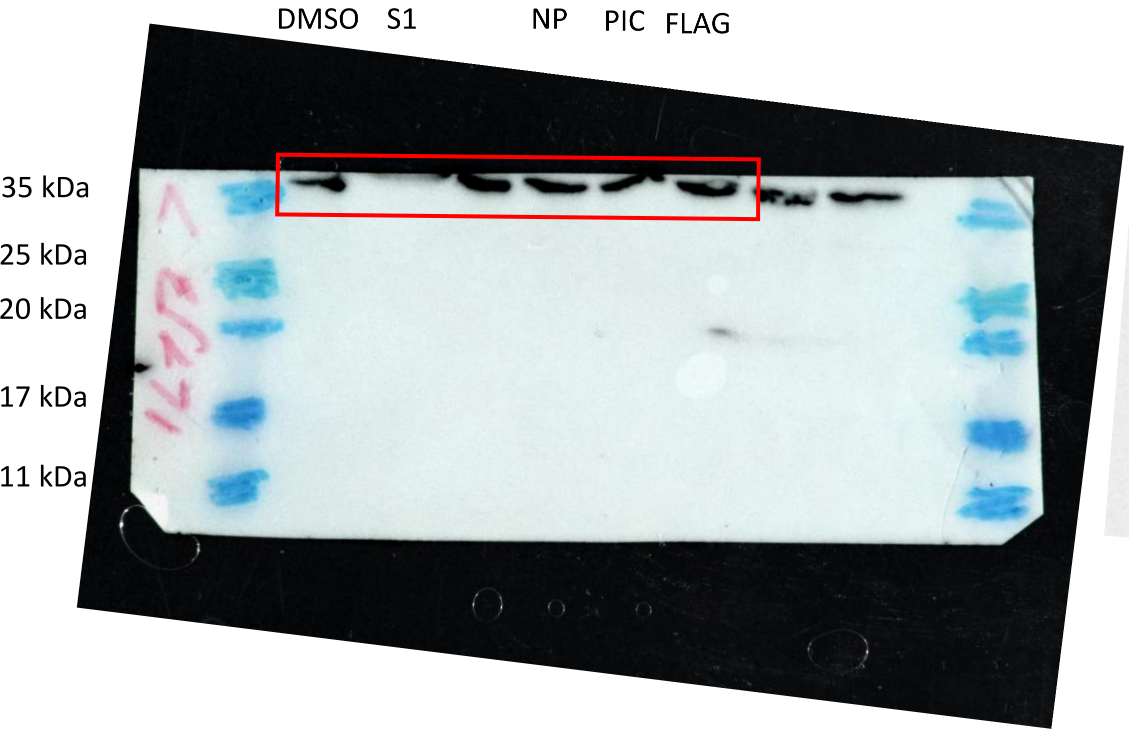

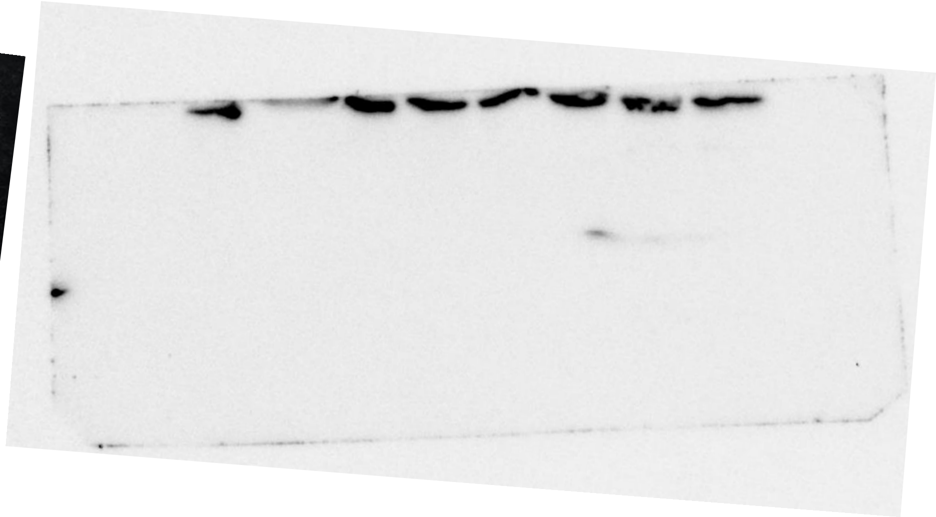

GAPDH (35 kDa) (FF26A/F9 BioLegend)

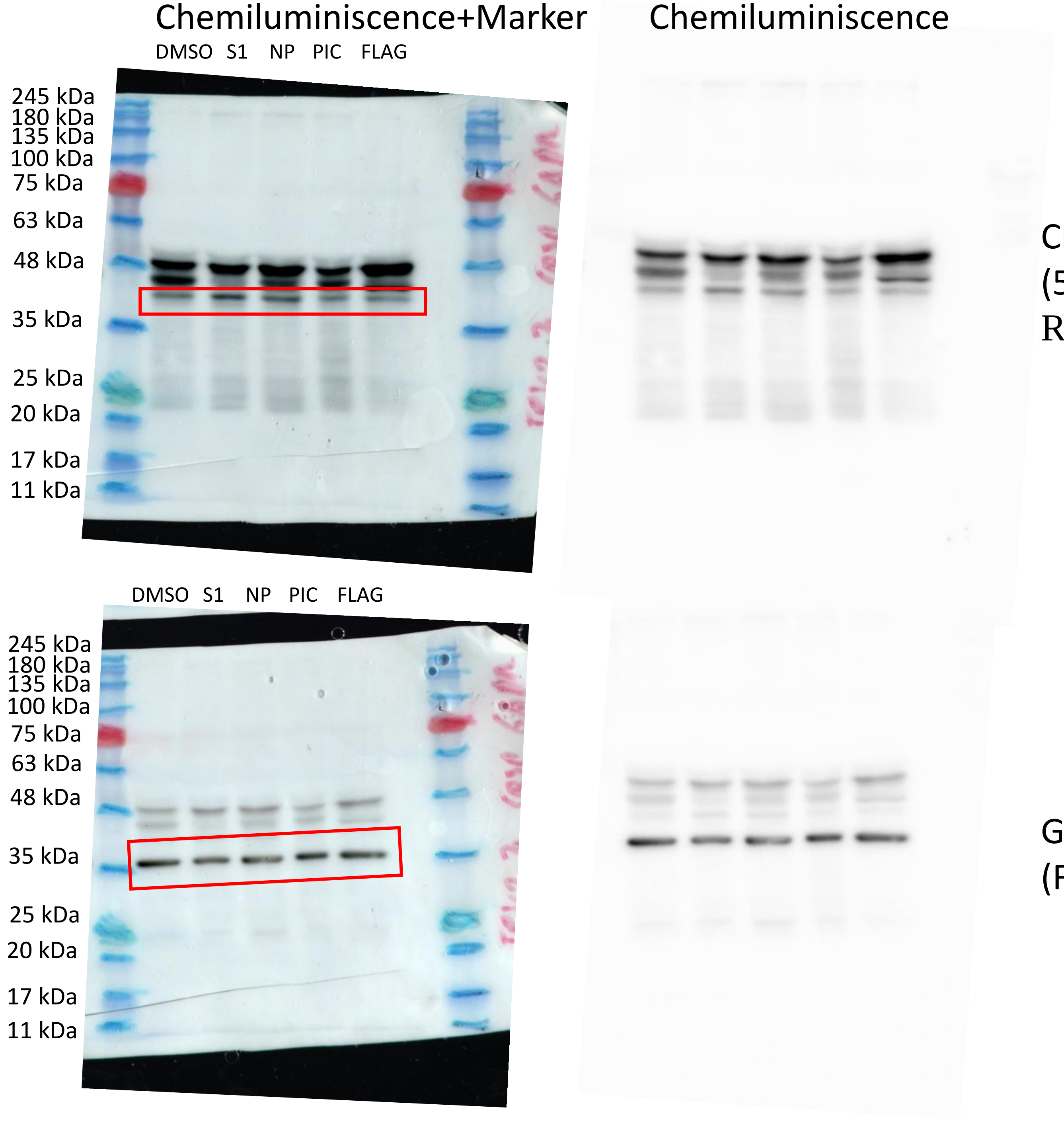

Caspase-1 and GAPDH included in Main figure 2C analysis

Cleaved Caspase-1 (52 kDa) (AF6215; R&D Systems)

GAPDH (35 kDa) (FF26A/F9 BioLegend)

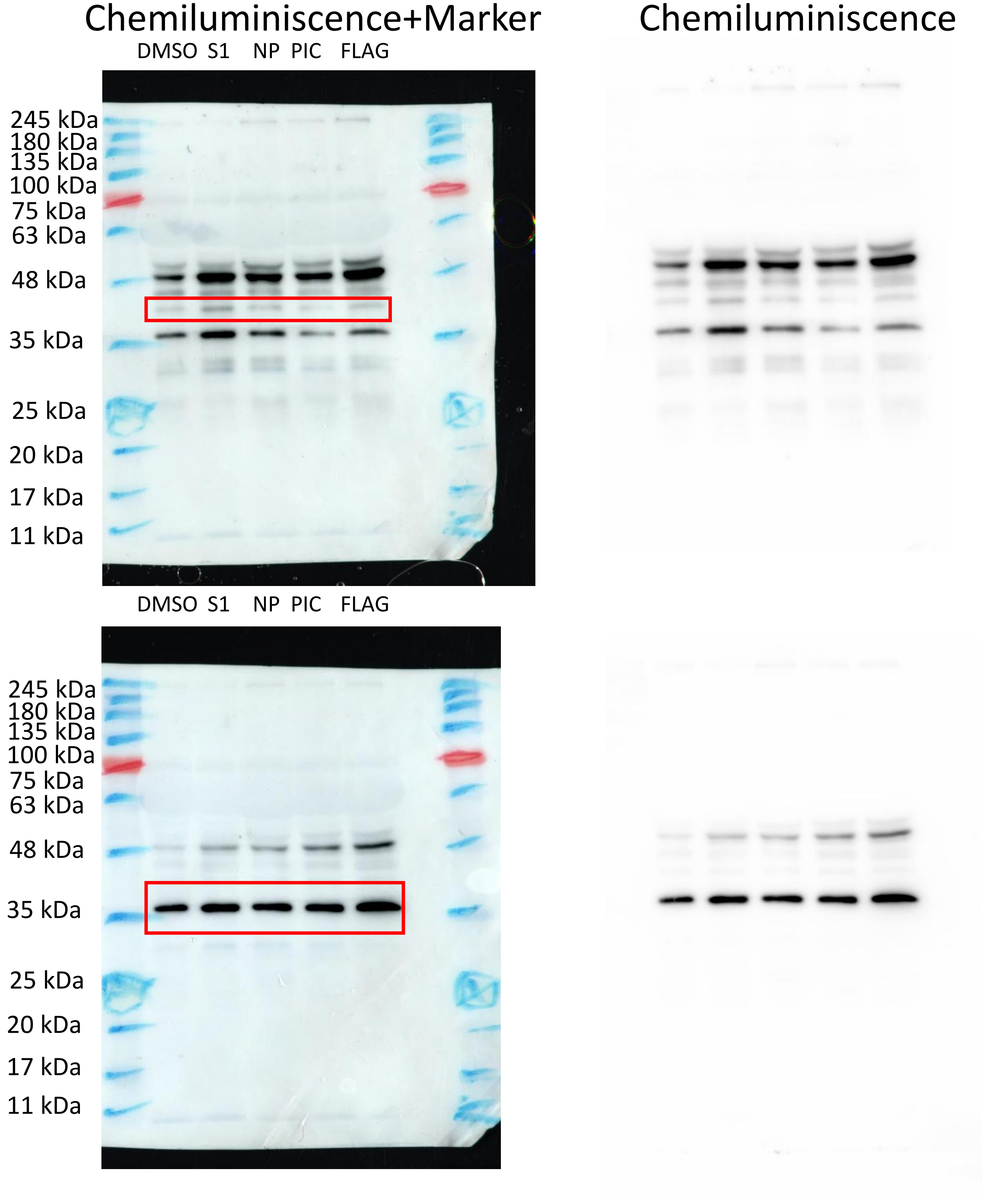

Caspase-1 and GAPDH included in Main figure 2C analysis

Cleaved Caspase-1 (52 kDa) (AF6215; R&D Systems)

GAPDH (35 kDa) (FF26A/F9 BioLegend)

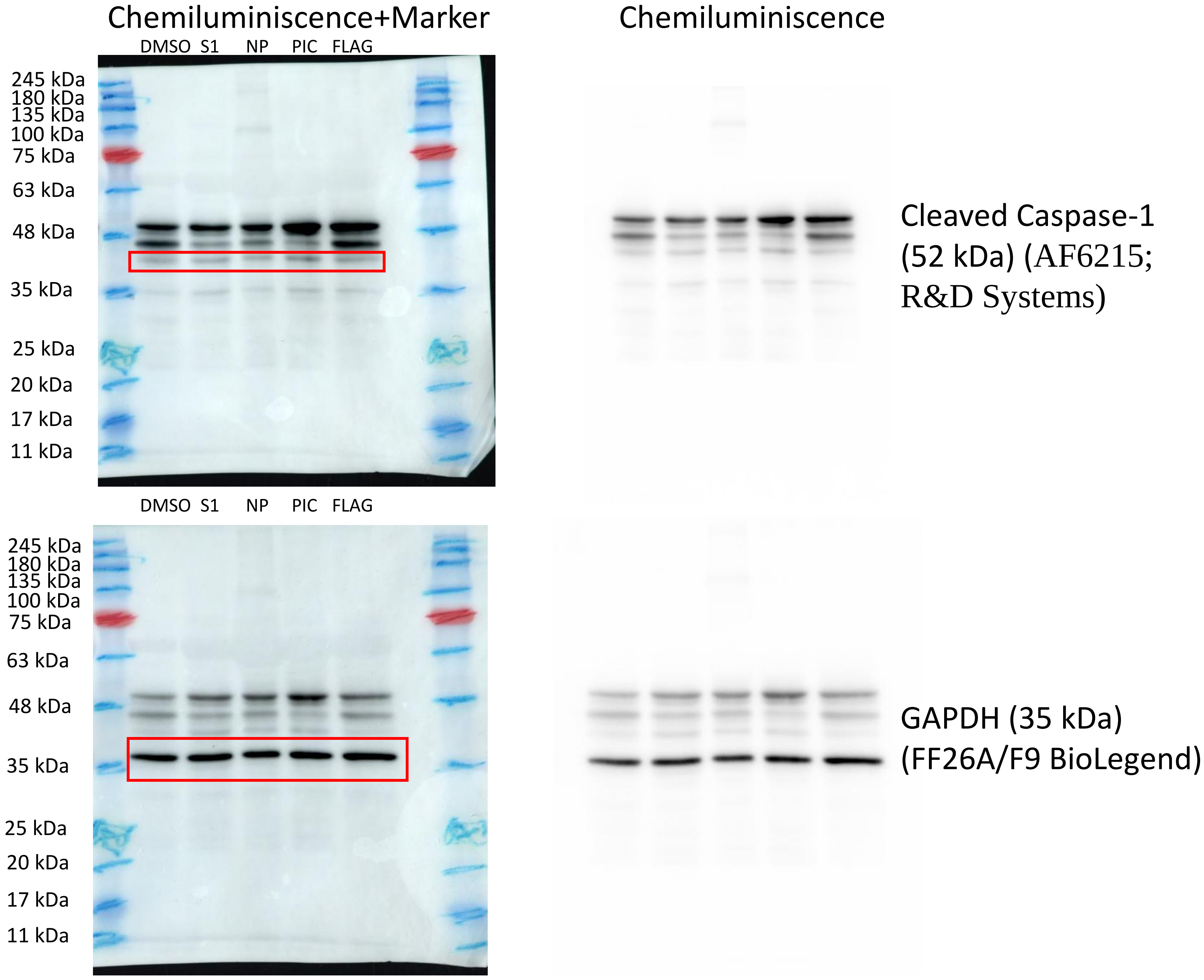

Caspase-1 and GAPDH included in Main figure 2C analysis

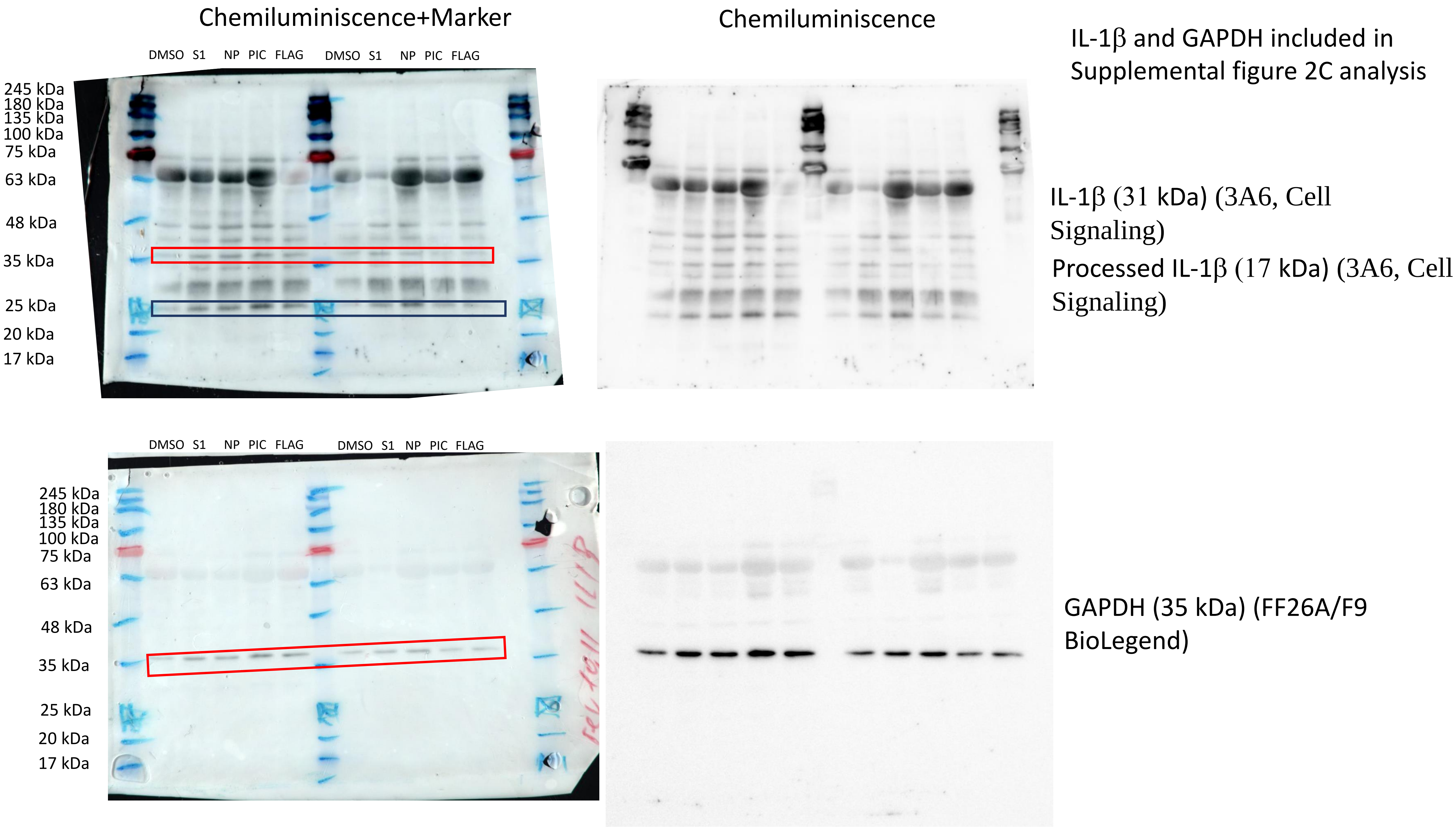
Chemiluminiscence+Marker
Chemiluminiscence
IL-1β and GAPDH included in Supplemental figure 2C analysis
DMSO S1 NP PIC FLAG DMSO S1 NP PIC FLAG
245 kDa
180 kDa
135 kDa
100 kDa
75 kDa
63 kDa
48 kDa
35 kDa
25 kDa
20 kDa
17 kDa
IL-1β (31 kDa) (3A6, Cell Signaling)
Processed IL-1β (17 kDa) (3A6, Cell Signaling)
DMSO S1 NP PIC FLAG DMSO S1 NP PIC FLAG
245 kDa
180 kDa
135 kDa
100 kDa
75 kDa
63 kDa
48 kDa
35 kDa
25 kDa
20 kDa
17 kDa
GAPDH (35 kDa) (FF26A/F9 BioLegend)

IL-1β and GAPDH included in Supplemental figure 2C analysis

Chemiluminiscence+Marker

Chemiluminiscence

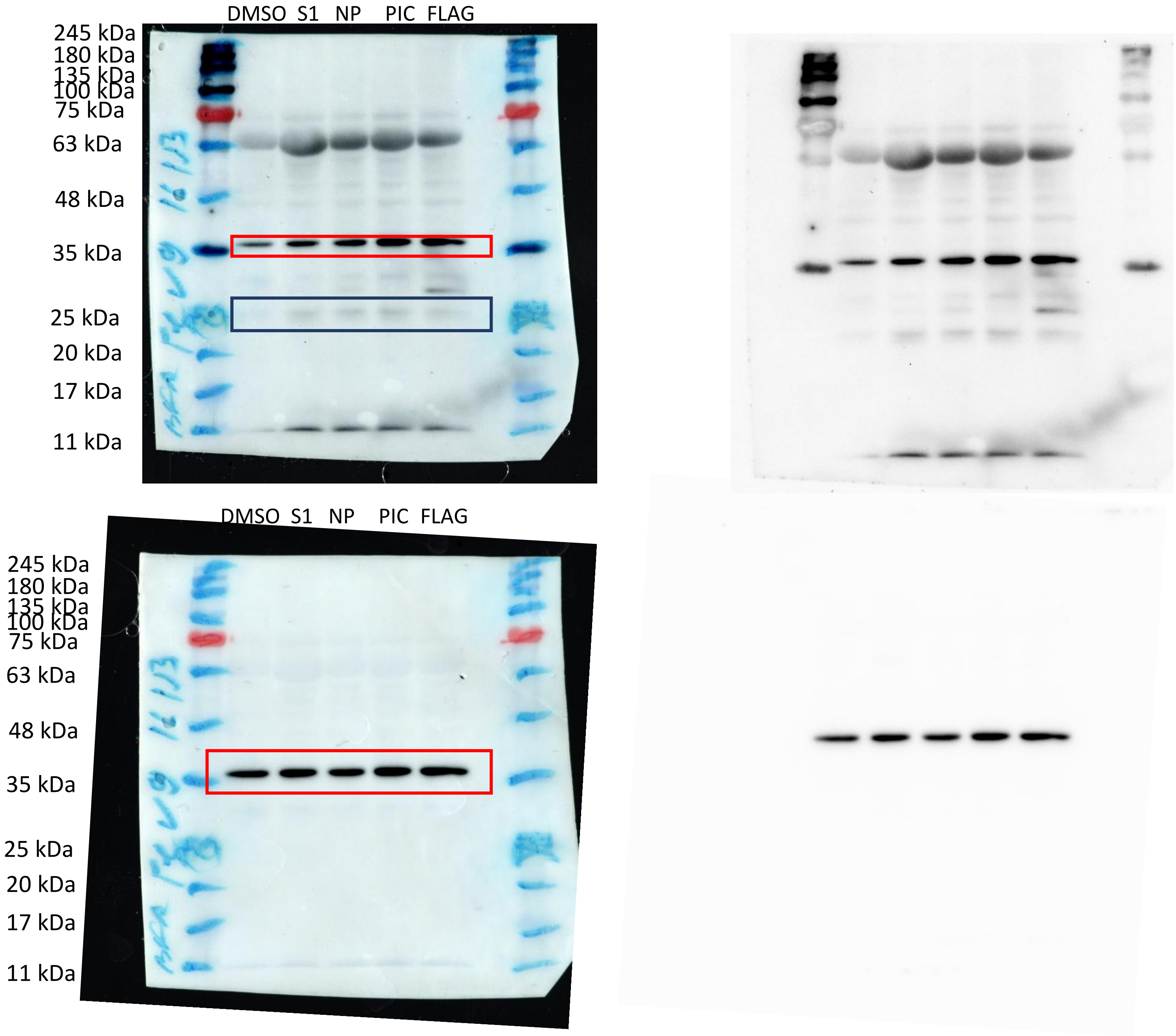

IL-1β (31 kDa) (3A6, Cell Signaling)

Processed IL-1β (17 kDa) (3A6, Cell Signaling)

GAPDH (35 kDa) (FF26A/F9 BioLegend)

Gasdermin D and GAPDH included in Supplemental figure 2C analysis

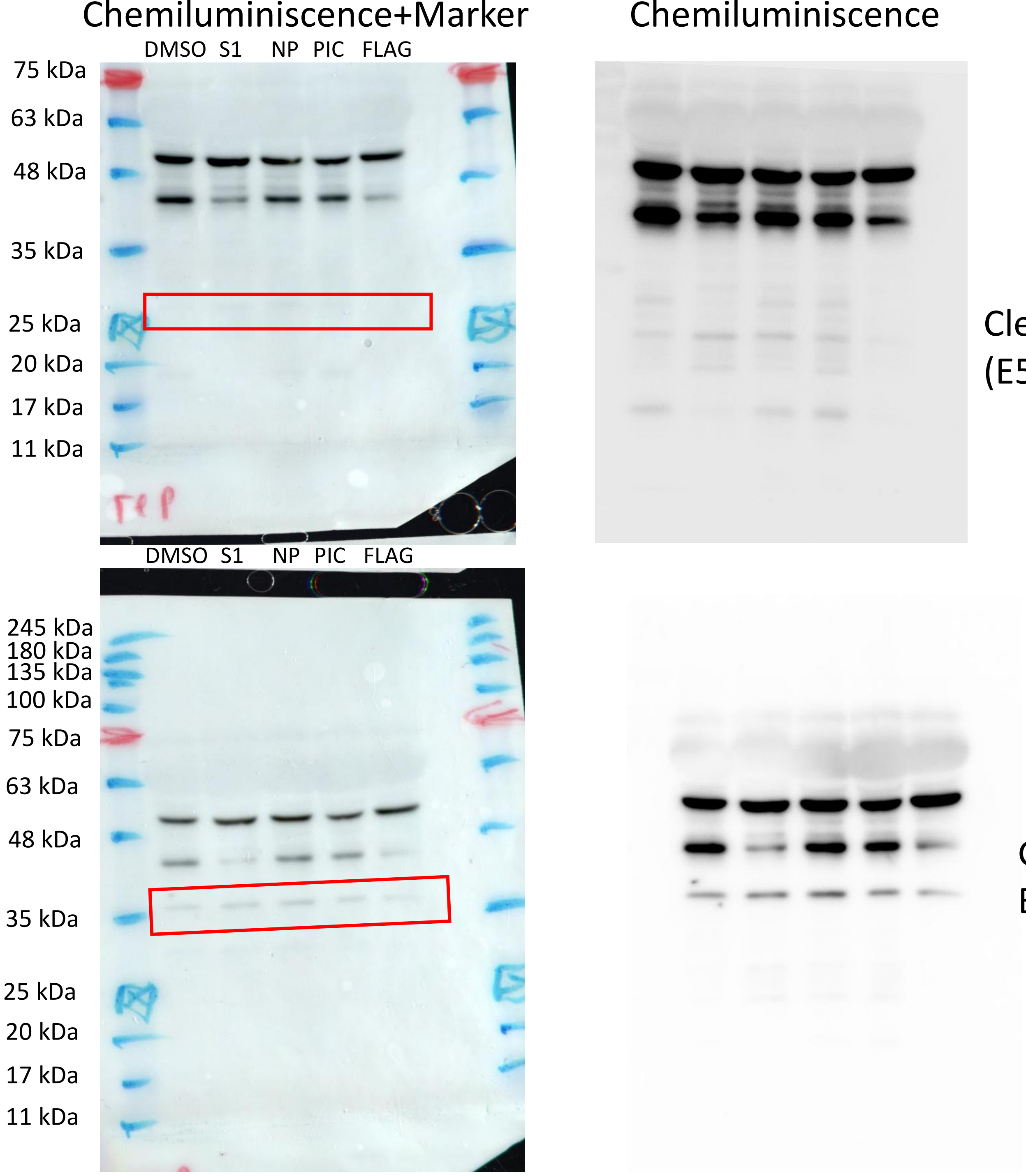

Cleaved Gasdermin D (21 kDa) (E5O4N, Cell Signaling)

GAPDH (35 kDa) (FF26A/F9 BioLegend)

Chemiluminiscence+Marker

Chemiluminiscence

Gasdermin D and GAPDH included in Supplemental figure 2C analysis

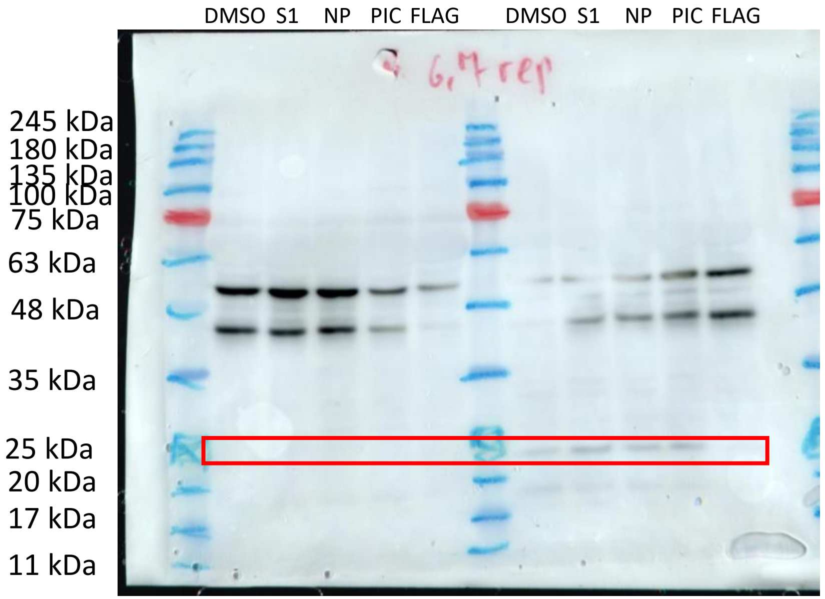

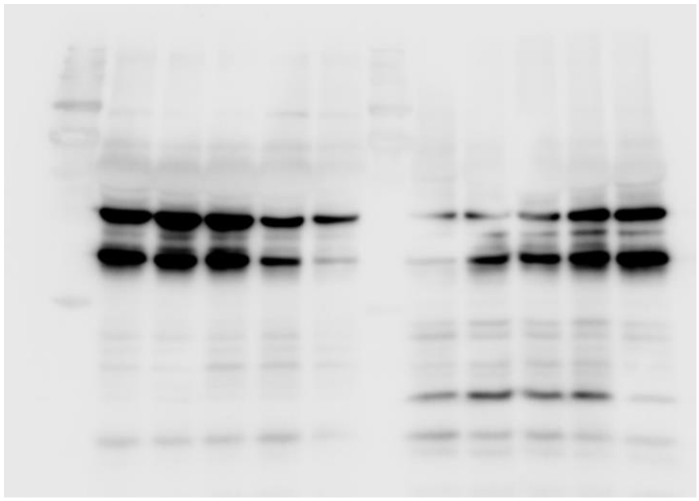

Cleaved Gasdermin D (21 kDa) (E5O4N, Cell Signaling)

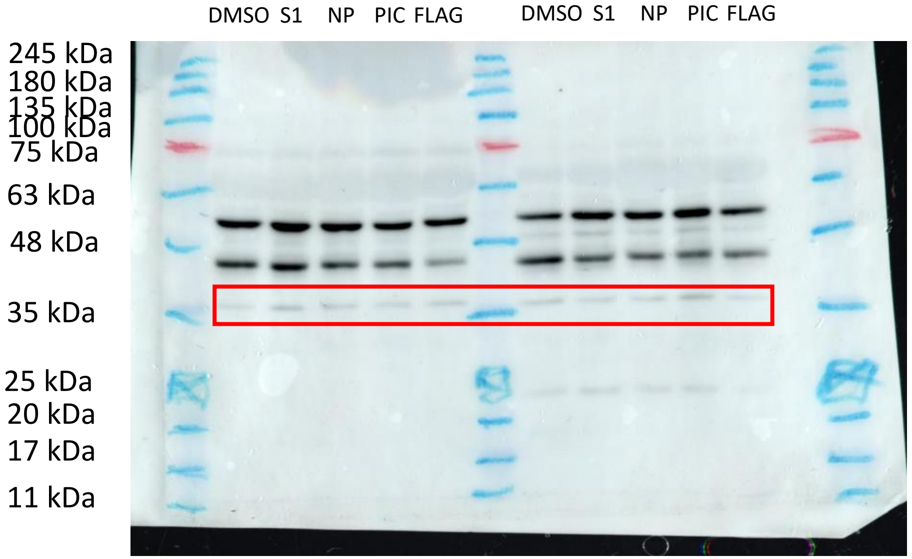

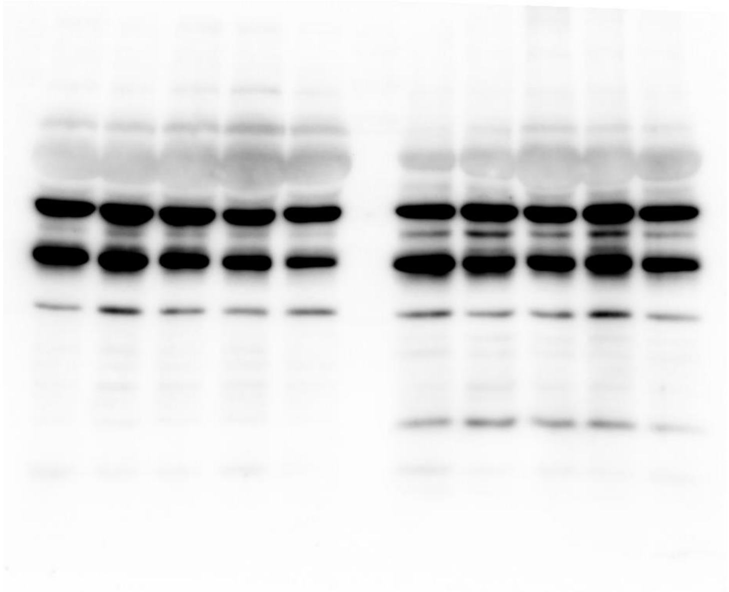

GAPDH (35 kDa) (FF26A/F9 BioLegend)
