# Supplementary material for: NFκB and NLRP3/NLRC4 inflammasomes regulate differentiation, activation and functional properties of monocytes in response to distinct SARS-CoV-2 proteins
Source: Nat Commun. 2024 Mar 7;15:2100. doi: 10.1038/s41467-024-46322-8 (PMC10920883; doi:10.1038/s41467-024-46322-8)
Supplement: Supplementary file 3 — Reporting Summary [file 41467_2024_46322_MOESM3_ESM.pdf]

nature portfolio

Corresponding author(s): EMG
Last updated by author(s): Jan 18, 2024

Reporting Summary

Nature Portfolio wishes to improve the reproducibility of the work that we publish. This form provides structure for consistency and transparency in reporting. For further information on Nature Portfolio policies, see our Editorial Policies and the Editorial Policy Checklist.

Statistics

For all statistical analyses, confirm that the following items are present in the figure legend, table legend, main text, or Methods section.

n/a	Confirmed	
☐	☒	The exact sample size (*n*) for each experimental group/condition, given as a discrete number and unit of measurement
☐	☒	A statement on whether measurements were taken from distinct samples or whether the same sample was measured repeatedly
☐	☒	The statistical test(s) used AND whether they are one- or two-sided<br>*Only common tests should be described solely by name; describe more complex techniques in the Methods section.*
☒	☐	A description of all covariates tested
☐	☒	A description of any assumptions or corrections, such as tests of normality and adjustment for multiple comparisons
☐	☒	A full description of the statistical parameters including central tendency (e.g. means) or other basic estimates (e.g. regression coefficient) AND variation (e.g. standard deviation) or associated estimates of uncertainty (e.g. confidence intervals)
☐	☒	For null hypothesis testing, the test statistic (e.g. *F*, *t*, *r*) with confidence intervals, effect sizes, degrees of freedom and *P* value noted<br>*Give P values as exact values whenever suitable.*
☒	☐	For Bayesian analysis, information on the choice of priors and Markov chain Monte Carlo settings
☒	☐	For hierarchical and complex designs, identification of the appropriate level for tests and full reporting of outcomes
☐	☒	Estimates of effect sizes (e.g. Cohen's *d*, Pearson's *r*), indicating how they were calculated

Our web collection on statistics for biologists contains articles on many of the points above.

Software and code

Policy information about availability of computer code

Data collection	Flow Cytometry data was obtained with BD FACSDiva Software (v9.0) and BD FACSuite Software (v1.5.0.925), qPCR data was obtained with StepOne V2.3 (Applied Biosystems) Microscopy data was obtained with Leica software, Chemiluminiscence data from Western Blot assays was obtained with IQ800 V1.2.0 Software
Data analysis	Flow Cytometry data was analyzed with FlowJo Software V10.7., qPCR data was analyzed with StepOne V2.3 (Applied Biosystems) Microscopy and Chemiluminiscence data from Western Blot assays was analyzed with ImageJ Software. Statistical analysis was performed in GraphPad Prism software (v8.0.0.)

For manuscripts utilizing custom algorithms or software that are central to the research but not yet described in published literature, software must be made available to editors and reviewers. We strongly encourage code deposition in a community repository (e.g. GitHub). See the Nature Portfolio guidelines for submitting code & software for further information.

Data

Policy information about availability of data

All manuscripts must include a data availability statement. This statement should provide the following information, where applicable:

- Accession codes, unique identifiers, or web links for publicly available datasets
- A description of any restrictions on data availability
- For clinical datasets or third party data, please ensure that the statement adheres to our policy

The source data for the analyses shown in this manuscript have been provided in the submission. Any additional experimental data used for the analyses that

support the findings of this study are available upon request to the corresponding author as specified in the Data avalaibility statement from the Methods section. The source data or the clinical data not have been uploaded to any repository.

Research involving human participants, their data, or biological material

Policy information about studies with human participants or human data. See also policy information about sex, gender (identity/presentation), and sexual orientation and race, ethnicity and racism.

Reporting on sex and gender	Sex and gender of those participants in which such information was available has been included in the Supplemental Tables 1 and 2.
Reporting on race, ethnicity, or other socially relevant groupings	There is no information regarding race or ethnicity or other socially relevant groups collected in our study
Population characteristics	The median age of our cohort is 69 years in the case of all COVID-19 samples. The comorbidites registered are events related to cardiovascular risk events and one respiratory disease like asthma. The majority of treatments administered to COVID-19 patients were related to antinflammatory, biological and antiviral drugs. This information is included in the Supplemental Table 1,2 and 3 and was not used to make comparisons between different patient groups.
Recruitment	The clinical criteria used to select COVID-19 patients used was hospitalized individuals clasified as severe and critical. Non COVID-19 lung control samples with similar demographic characteristics were recruited as BAL controls.
Ethics oversight	The patients were recruited for the study using informed consent (reference 4381) approved by the bioethical committee from Hospital Universitario La Princesa de Madrid following principles of the Helsinki declaration.

Note that full information on the approval of the study protocol must also be provided in the manuscript.

Field-specific reporting

Please select the one below that is the best fit for your research. If you are not sure, read the appropriate sections before making your selection.

☒ Life sciences ☐ Behavioural & social sciences ☐ Ecological, evolutionary & environmental sciences

For a reference copy of the document with all sections, see nature.com/documents/nr-reporting-summary-flat.pdf

Life sciences study design

All studies must disclose on these points even when the disclosure is negative.

Sample size	no previous statistical power calculation was applied to the study. The number of COVID-19 PBMC and BAL amples used were highly dependent on the availability of this special type of samples
Data exclusions	Data exclusion criteria has been included in the methods section of our revised manuscript.
Replication	Multiple biological replicates were included on each analysis and number of different samples used have been detailed for all analyses in each figure legend from the revised manuscript.
Randomization	Samples were not randomized for this study since the study was already focused in a specific group of critical COVID-19 severity and we did not perform a comparison with other study groups.
Blinding	A blinding strategy was not applied for most of the analyses performed in the study. Correlation network analyses with clinical parameters were blinded since we did not previously select samples based on time of hospitalization, exitus, plasma cytokine levels, etc. However data acquisition was performed by technicians that were not familiarized with the different conditions and therefore was unbiased.

Reporting for specific materials, systems and methods

We require information from authors about some types of materials, experimental systems and methods used in many studies. Here, indicate whether each material, system or method listed is relevant to your study. If you are not sure if a list item applies to your research, read the appropriate section before selecting a response.

Materials & experimental systems

n/a	Involved in the study
☐	☒ Antibodies
☒	☐ Eukaryotic cell lines
☒	☐ Palaeontology and archaeology
☒	☐ Animals and other organisms
☐	☒ Clinical data
☒	☐ Dual use research of concern
☒	☐ Plants

Methods

n/a	Involved in the study
☒	☐ ChIP-seq
☐	☒ Flow cytometry
☒	☐ MRI-based neuroimaging

Antibodies

Antibodies used	Detailed information for all antibodies and dilutions used for flow cytometry and immunofluorescence analyses have been included both in the Supplemental Tables 4 and 5 and in the Method section from our revised manuscript.
Validation	All antibodies used for the study have been obtained from commercial sources and were previously validated for the particular experimental application used in the study. This information is included through the catalog number and clone information included for each antibody in the supplemental tables 4 and 5.

Clinical data

Policy information about clinical studies

All manuscripts should comply with the ICMJEguidelines for publication of clinical research and a completedCONSORT checklist must be included with all submissions.

Clinical trial registration	n/a
Study protocol	n/a
Data collection	n/a
Outcomes	n/a

Plants

Seed stocks	n/a
Novel plant genotypes	n/a
Authentication	n/a

Flow Cytometry

Plots

Confirm that:

☒ The axis labels state the marker and fluorochrome used (e.g. CD4-FITC).

☒ The axis scales are clearly visible. Include numbers along axes only for bottom left plot of group (a 'group' is an analysis of identical markers).

☒ All plots are contour plots with outliers or pseudocolor plots.

☒ A numerical value for number of cells or percentage (with statistics) is provided.

Methodology

Sample preparation	Information for sample preparations are detailed in the Methods section
Instrument	The flow cytometer used has been detailed in the Methods section

Software	Flowjo software was used for flow cytometry data analysis. This information is included in the methods section from our manuscript.
Cell population abundance	Proportions and purity of cell populations have been provided
Gating strategy	Gating strategy has been represented in supplemental figures 1, 4 and 5 from our revised manuscript

☒ Tick this box to confirm that a figure exemplifying the gating strategy is provided in the Supplementary Information.
